# Intracranial EEG referencing for large-scale category-selective mapping in the human ventral occipito-temporal cortex

**DOI:** 10.1162/imag_a_00479

**Published:** 2025-02-24

**Authors:** Simen Hagen, Corentin Jacques, Radu Ranta, Laurent Koessler, Louis Maillard, Sophie Colnat-Coulbois, Bruno Rossion, Jacques Jonas

**Affiliations:** Université de Lorraine, CNRS, IMoPA, Nancy, France; Université de Lorraine, CNRS, CRAN, Nancy, France; Université de Lorraine, CHRU-Nancy, Service de Neurologie, Nancy, France; Université de Lorraine, CHRU-Nancy, Service de Neurochirurgie, Nancy, France

**Keywords:** iEEG, reference montage, human face recognition, frequency-tagging

## Abstract

Intracranial EEG (iEEG) is increasingly used in many fields of human cognitive neuroscience since it offers a unique opportunity to directly record brain activity from awake humans at a high spatial and temporal resolution. However, little is known about the influence of the reference montage on the spatial and temporal characteristics of iEEG activity. Here, we compare the spatial and temporal profiles of neural activity for five reference montages (scalp reference, common average, zero reference, local Bipolar, and Laplacian) applied to a large dataset of depth electrodes (StereoElectroEncephaloGraphy, SEEG) recordings across the human ventral occipito-temporal cortex (VOTC, N individual brains = 77). Frequency-tagging is used for objective identification and quantification of both low- (<30 Hz) and high-frequency (40–160 Hz) face-selective neural activity. For low-frequency responses, similar spatial distributions and time-courses of significant face-selective contacts and of face-selective amplitudes are found across the five reference montages, although the latter two local reference montages enhance face selectivity along the fusiform gyrus until the anterior temporal lobe. However, they also reduce the right hemisphere dominance, a hallmark of face-selective neural activity, and increase the number of significant contacts in the white matter. For high-frequency responses, similar spatial distributions and time-courses of significant face-selective contacts and of face-selective amplitudes are found for all references, except for the scalp reference (SCA), which enhances face selectivity in lateral and medial regions of the anterior VOTC. However, SCA also increases the number of significant contacts in the white matter. Thus, specificities of each electrode montage should be considered before choosing an iEEG reference, according to the research question, the anatomical region, the type of analyses, and the responses frequency range.

## 1 Introduction

Electroencephalographic recording from intracranial electrodes implanted in epileptic patients (intracranial EEG or iEEG) is a long-standing and increasingly used approach to understand the neural basis of sensory, motor, and cognitive functions in humans (Axmacher, 2023). Compared with non-invasive methods (e.g., EEG, MEG, fMRI), iEEG provides direct recording of neural activity with both high spatial and temporal resolution.

An important methodological issue facing iEEG, and other electrophysiological measures, is the use of a reference electrode that is as neutral as possible, the so-called reference problem (e.g.,Joyce & Rossion, 2005;Kayser & Tenke, 2010;Nunez & Srinivasan, 2006;Regan, 1989). Specifically, electrophysiological activity reflects an electric potential difference (voltage) between a recording site, the active site, and another site, the reference site. The ideal situation is usually thought of as having the active site close to the source of neural activity of interest (e.g., elicited by an internal or external stimulus), and the reference site remote from any major neural activity (i.e., neutral or silent). The difference of potential between the two sites would then essentially reflect the measure of brain activity at the active site. However, in reality, there are numerous factors that influence the magnitude and morphology of the electrophysiological signal measured at the active site, which include the complex interaction between the location of the active and the reference site, the location and distance of the neural source(s), and the orientation of the electrical fields related to the pattern of cortical folding generating the electrical fields. This issue is well known for scalp EEG studies, in which the reference (electrode) problem has been studied since the 1950s: studies show that different reference montages can drastically modify the shape, amplitude, and localization of brain activity on the scalp (Joyce & Rossion, 2005;Kayser & Tenke, 2010;Nunez & Srinivasan, 2006;Regan, 1989;Yao et al., 2019). Thus, to achieve a spatially coherent measure, it is important to use a reference montage that mainly reflects local activity at the active site, maximizing the signals of interest and minimizing noise contamination from the reference.

In comparison with scalp EEG, little is known about how the reference montage impacts neural activity measured directly in the gray and white matter with iEEG (e.g.,Mercier et al., 2022;Parish et al., 2023). Notably, iEEG studies typically rely on a wide variety of reference montages: a physical site, either on the scalp (SCA), inside the white matter or in the skull; a common average reference (CAR) using the mean signal over all recording sites; or else local reference methods using a site in the immediate vicinity of the active site (e.g., its adjacent site, i.e., local Bipolar reference (BIP), or the mean of the two adjacent sites, i.e., Laplacian reference (LAP)).

Notably, even within the same field of study, various reference methods are used. For example, among 60 iEEG studies that reported brain responses to the presentation of human face stimuli over the last three decades (between 1994 and 2024), 24 (40%) used a physical electrode, 18 (30%) used a CAR, and 5 (8%) a local reference method (BIP) (14 not reported (23%); seeSupplementary Table S1for a list of all studies with their reference montage). Yet, iEEG studies evaluating the effect of reference montages (usually not taking into account whether the cortical tissue is functionally relevant to the task-related recordings) show that, compared with CAR or physical site references, local references minimize inter-channel correlations, maximize task-related correlations, and increase signal-behavior decoding performance (Li et al., 2018;S. Liu et al., 2021;Y. Liu et al., 2015;McCarty et al., 2022;Meisler et al., 2019; see alsoMercier et al., 2017). Nevertheless, the effect of reference montage on the spatial distribution and temporal profile of functional neural activity recorded on a wide functionally relevant cortical surface remains largely unknown.

Here we address the issue of the reference montage for iEEG studies, comparing five reference montages. Our contribution aims at providing valuable information for iEEG studies in general and is original at several levels. First, we rely on a particularly large sample of iEEG recordings from depth electrodes (SEEG), that is, 77 individual brains, yielding a rich dataset to address the reference issue. Second, we focus on a specific, yet densely sampled and extensive cortical territory, the ventral occipito-temporal cortex (VOTC), running from the occipital pole to the temporal pole over many distinct anatomical regions, in both hemispheres. Third, rather than measuring general unspecific responses, we focus on isolated functional neural activity, namely category-selective activity recorded to human faces, that is, highly familiar, ecologically valid, stimuli recruiting a large portion of the human VOTC (Duchaine & Yovel, 2015;Gao et al., 2018;Grill-Spector et al., 2017;Haxby et al., 2000;Sergent et al., 1992). Since face-selective activity is typically recorded bilaterally with a right hemisphere advantage (Gao et al., 2018;Sergent et al., 1992; seeRossion & Lochy, 2022) and with the largest amplitude in well-known discrete VOTC regions, this also provides us with the opportunity to test for the effect of reference electrode montage on hemispheric lateralization and localization. Fourth, to overcome the challenge of comparing across multiple reference montages with rich iEEG signals, we rely on a powerful frequency-tagging method (Norcia et al., 2015;Regan, 1989;Rossion et al., 2018) that provides objective identification (i.e., at the stimulated frequency) and quantification (in the frequency domain) of neural activity separately for both low- and high-frequency ranges (seeFig. 1).

**Fig. 1.**
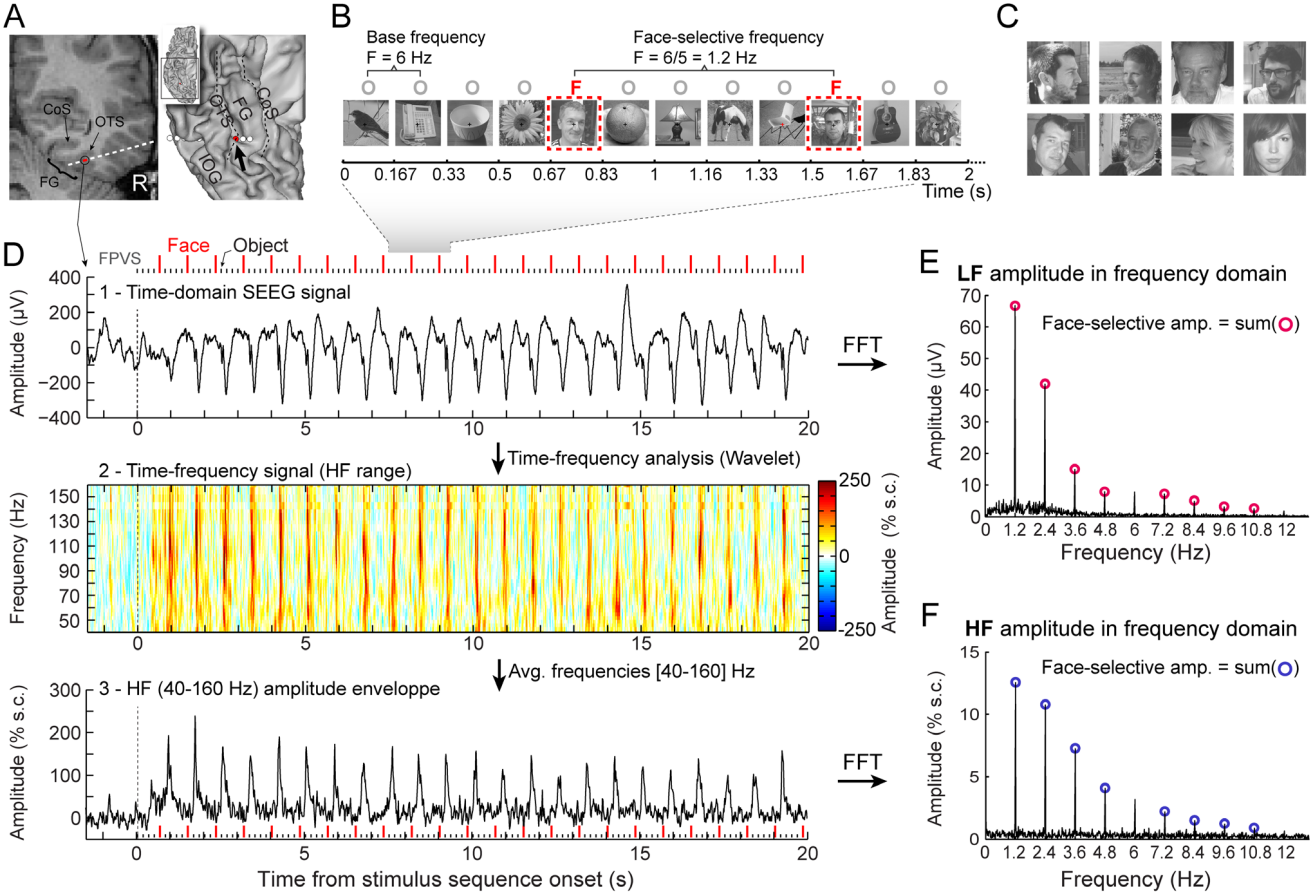
Recording and quantifying SEEG low-frequency (LF) and high-frequency (HF) face-selective signals in the VOTC (adapted fromJacques et al., 2022). (A) Left: Coronal slice of an example depth (SEEG) electrode implanted in the right VOTC of an individual participant. Right: the same SEEG electrode array is shown on the reconstructed white matter surface of the participant (ventral view of the right hemisphere). Intracerebral electrode arrays consist of 8–15 contiguous recording contacts (small white rectangles in the coronal slice, white circles on the 3D surface) spread along the electrode length. Electrodes penetrate both gyral and sulcal cortical tissues. Here the electrode extends from the fusiform gyrus to the middle temporal gyrus. The recording contact located at the junction between the lateral fusiform gyrus and occipito-temporal sulcus and where the signal shown in panels D–F is measured is highlighted in red (left: red rectangles surrounded by a circle; right: red circles, see the black arrow). COS: collateral sulcus; OTS: occipito-temporal sulcus; FG: fusiform gyrus; IOG: inferior occipital gyrus. (B) The fast periodic visual stimulation (FPVS) (or frequency-tagging) paradigm used to quantify face-selective neural activity (originally fromRossion et al., 2015; see e.g.,Jacques et al., 2016;Rossion et al., 2018): natural images of non-face objects are presented by sinusoidal contrast modulation at a rate of six stimuli per second (6 Hz) with highly variable face images presented every five stimuli. Common neural activity to faces and non-face objects is expressed at 6 Hz and harmonics in the iEEG signals, so that any selective (i.e., differential) activity elicited reliably by face stimuli appears at the frequency of 6/5 = 1.2 Hz. Each stimulation sequence lasts for 70 s (2 s shown here). (C) Representative examples of natural face images used in the study (actual images not shown for copyright reasons). (D) Top: example raw intracranial EEG time-domain signal measured at the recording contact shown in panel (A). The signal is shown from –1.5 to 20 s relative to the onset of a stimulation sequence. The time series displayed is an average of two sequences. Above the time series, red vertical ticks indicate the appearance of face image in the sequence every 0.835 s (i.e., every 5^th^image at 6 Hz) and small black vertical ticks indicate the appearance of non-face objects every 0.167 s. Example images shown in each sequence are shown in panel B. Middle: a time-frequency representation of the SEEG data in the HF range (40–160 Hz) is obtained with a wavelet transform. The plot shows the percent signal change at each frequency relative to a pre-stimulus baseline period (–1.6 s to –0.3 s). This highlights distinct periodic burst of HF activity occurring at the frequency of face stimulation (i.e., 1.2 Hz) after the start of the stimulation sequence. Bottom: The modulation of HF amplitude over time (i.e., HF amplitude envelope) is obtained by averaging time-frequency signals across the 40–160 Hz frequency range. Red vertical ticks indicate the appearance of face images in the sequence. (E) LF face-selective amplitude is quantified by transforming the time-domain iEEG signal to the frequency domain (Fast Fourier Transform, FFT) and summing amplitudes of the signal at 12 harmonics of the frequency of face stimulation (1.2, 2.4, 3.6, 4.8, … Hz, excluding harmonics of the 6 Hz base stimulation rate). (F) HF face-selective amplitude is quantified in the same manner as for LF (panel E) with FFT applied to the HF amplitude envelope.

## 2 Methods

### 2.1 Participants

The study included 77 patients (33 females, mean age: 31.7 ± 8.5 years, 69 right handed, 1 ambidextrous, 7 left handed) undergoing clinical intracerebral evaluation with depth electrodes (StereoElectroEncephaloGraphy, SEEG) for refractory partial epilepsy, studied in the Epilepsy Unit of the University Hospital of Nancy. Patients gave written informed consent to participate in the study which was conducted according to the Declaration of Helsinki and approved by the ethical board of the CHRU-Nancy. They were included in the study if they had at least one intracerebral electrode implanted in the VOTC.

### 2.2 Intracerebral electrode implantation and recording

The methods are the same as in previous studies (Hagen et al., 2020;Jonas et al., 2016). Intracerebral electrodes were stereotactically implanted within the participants’ brains (Fig. 1A) for clinical purposes, that is, to delineate their seizure onset zones and to functionally map the surrounding cortex in the perspective of an epilepsy surgery (Bédos-Ulvin et al., 2017). Each 0.8 mm diameter intracerebral electrode contains 8–15 independent recording contacts of 2 mm in length separated by 1.5 mm from edge to edge (for details about the electrode implantation procedure; seeSalado et al., 2018).

Intracerebral EEG was sampled at a 512 Hz with a 256-channel amplifier (Micromed) and recorded using a single electrode reference on the scalp (SCA), that is, specifically a midline prefrontal scalp electrode (FPz).

### 2.3 Fast periodic visual stimulation (FPVS) paradigm

#### 2.3.1 Stimuli

We used 200 grayscale natural images of various non-face objects (from 14 non-face categories: cats [n = 9], dogs [n = 5], horses [n = 5], birds [n = 24], flowers [n = 15], fruits [n = 28], vegetables [n = 21], houseplants [n = 15], phones [n = 13], chairs [n = 15], cameras [n = 6], dishes [n = 15], guitars [n = 15], lamps [n = 14]), and 50 grayscale natural images of faces (seeFig. 1B, Cfor examples of stimuli; all images taken from a paradigm validated in scalp EEG and iEEG studies; e.g.,Rossion et al., 2015,^2018^). Each image contained an unsegmented object or a face, which were located near the center. Faces and objects varied substantially in terms of size, viewpoint, lighting conditions, and background across images (seeRossion et al., 2015). Images were equalized for mean pixel luminance and contrast (i.e., standard deviation across pixels).

#### 2.3.2 Experimental procedure

Participants viewed continuous sequences of natural images of objects presented at a fast rate of 6 Hz through sinusoidal contrast modulation, in which faces were presented periodically as every 5^th^stimulus so that the frequency of face presentation was 1.2 Hz (i.e., 6 Hz/5) (Fig. 1B). All images were randomly selected from their respective categories. A sequence lasted 70 s: 66 s of stimulation at full-contrast flanked by 2 s of fade-in and fade-out, where contrast gradually increased or decreased, respectively. During the sequences, participants performed an orthogonal color-change detection task on the fixation cross, where they were instructed to detect brief (500 ms) black to red changes. Sequences were repeated a minimum of two times (average sequences across patients: 2.8, range: 2–5).

Participants were not informed about the periodicity of the stimulation and were unaware of the objectives of the study. No participant had seizures in the 2 h preceding fast periodic visual stimulation (FPVS) recordings.

### 2.4 SEEG signal processing and analyses

#### 2.4.1 Referencing

Segments of SEEG corresponding to stimulation sequences were extracted (74 s segments, -2 s to +72 s). Each recorded epoch was acquired with a scalp reference montage (SCA). These datasets were subsequently referenced to four other montages: (1) a local Bipolar montage (BIP), where signals are referenced to the neighboring medial contact on the same electrode array; (2) a Laplacian montage (LAP), where signals are referenced to the average value of the two neighboring lateral and medial contacts on the same electrode; (3) a common average montage (CAR), where signals are referenced to the average amplitude of all contacts (Offner, 1950); (4) a “zero reference” montage (REF0), where signals are referenced to a virtual reference computed as a weighted average among the raw signals: REF0 = ∑(w_i*SCA_i)/N, where the weights w_i are computed using the semi-blind beamformer introduced in previous work (Hu et al., 2007;Ranta et al., 2010). This zero reference, developed to better determine and eliminate the reference signal from the electrode montage, has previously been validated with SEEG recordings at rest (Madhu et al., 2012).

#### 2.4.2 Low-frequency activity analysis

The 74 s data segments were cropped to contain an integer number of 1.2 Hz cycles (face oddball rate) beginning 2 s after the onset of the sequence (right at the end of the fade-in period) until approximately 65 s, that is, before stimulus fade-out (75 face cycles ≈ 63 s). Next, the recorded temporal sequences of voltage were averaged separately for each reference montage and contact. Averaging sequences in the time domain cancels out non-phase locked noise, and so increases the signal-to-noise ratio of the subsequent Fast Fourier Transform (FFT) (described below). No artifact correction was applied because (1) (S)EEG artifacts generate noise at frequencies that locate mostly outside of the frequencies of interest (1.2 Hz and associated harmonics) and, most importantly, the noise is broadband while the signal locates in narrow frequency bins due to the very high-frequency resolution of our approach (Regan, 1989;Rossion, 2014).

Subsequently, an FFT was applied to the full length of the cropped averaged time sequences for each reference montage. The amplitude spectra were extracted for all contacts by taking the modulus of the Fourier coefficients at each frequency bin normalized (by dividing) by half of the number of time samples in the time series. The long recording sequence resulted in a spectrum with a high-frequency resolution of 0.0159 Hz (1/63 s). No data segments were excluded from the analysis. No other processing was performed to the data.

The FPVS approach used here allows identifying and separating two distinct types of responses to the face stimuli: (1) a general visual response occurring at the base stimulation frequency and its harmonics (6 Hz, 12 Hz, 18 Hz, …), as well as (2) a face-selective response at the oddball stimulation frequency and its harmonics (1.2 Hz, 2.4 Hz, 3.6 Hz, …). Face-selective responses significantly above noise level at the face frequency and its harmonics for each reference montage were determined as follows: (1) the FFT spectrum was cropped into 4 segments centered at the face frequency and harmonics, from the 1^st^until the 4^th^(faces: 1.2 until 4.8 Hz), and surrounded by 25 neighboring bins on each side, corresponding to a frequency band of 0.8 Hz centered on the harmonics of interest; (2) the amplitude values in these 4 segments of FFT spectra were summed; and (3) the summed FFT spectrum was transformed into a Z-score. Z-scores were computed as the difference between the amplitude at the face frequency bin and the mean amplitude of 48 surrounding bins (25 bins on each side, excluding the 2 bins directly adjacent to the bin of interest, i.e., 48 bins) divided by the standard deviation of amplitudes in the corresponding 48 surrounding bins. A contact was considered as showing a face-selective response in each reference montage if the Z-score at the frequency bin of face stimulation exceeded 3.1 (i.e.,*p*< 0.001 one-tailed: signal > noise).

To extract the temporal profile for each reference montage, we (1) low-pass filtered the data in the temporal domain using a Butterworth bandpass filter (0–40 Hz), (2) resampled it to 500 Hz, (3) applied a notch filtered to remove the base rate signal (6 Hz and 4 harmonics, width = 1 Hz), (4) created epochs relative to the face stimulus onsets (-332 to 833 ms) (84 epochs per 70 s sequence), (5) averaged across the epochs created from step 4, (6) applied a baseline correction using 1 cycle prior to stimulus onset (167 ms), and (7) transformed all data to positive values to reflect contact activation and avoid the canceling of the responses with opposite polarities.

#### 2.4.3 High-frequency activity analysis

Starting from the 74 s segments of iEEG corresponding to stimulation sequences in each reference montage (74 s segments, –2 s to +72 s), variation in signal amplitude as a function of time and frequency was estimated by a Morlet wavelet transform (Jacques et al., 2022) applied on each SEEG segment from frequencies of 40–160 Hz (i.e., high-frequency broadband (HF)), in 2 Hz increments (Fig. 1). The number of cycles (i.e., central frequency) of the wavelet was adapted as a function of frequency from 4 cycles at the lowest frequency to 11 cycles at the highest frequency, with a constant standard deviation in the time domain of 0.15 s. The frequency bandwidth of the lowest wavelet (4 cycles at 40 Hz) was 25 Hz (full width at half maximum). Using these parameters, the lower boundary of the frequency bandwidth for the HF signal was 27.5 Hz (i.e., 40–25/2 Hz). The wavelet transform was computed on each time sample and the resulting amplitude envelope was resampled by a factor of 3 (i.e., to 170.7 Hz sampling rate) to save disk space and computation time. For each segment, amplitude was normalized across time and frequency to obtain the percent of signal change generated by the stimulus onset relative to the mean amplitude in a pre-stimulus time window (-1,600 to -300 ms relative to the onset of the stimulation sequence). This normalization step ensures that each frequency in the HF range contributes equally to the computed HF signal, despite the overall 1/f relationship between amplitude and frequency in EEG. The percent signal change was then averaged across frequencies (between 40 and 160 Hz) to obtain the time-varying HF amplitude envelope (Fig. 1D, bottom).

To determine significance, the 74 s segments-averaged-across-frequencies were averaged in the time domain (2 to 5 segments per participants). The resulting averaged segments were then cropped to contain an integer number of 1.2 Hz cycles (from 2 s after sequence onset to about 68 s). The frequency content of the HF envelope was obtained using an FFT (Fig. 1F). Significant face-selective responses in HF were detected based on the frequency spectra in the same way as for the low-frequency bands.

To extract temporal profiles, a notch filter was applied to the segments-averaged-across-frequencies (width = 1 Hz, number of harmonics = 4) to filter out the base response at 6 Hz and harmonics. The segments were then epoched relative to the face onsets (-332 to 600 ms), and the epochs were averaged.

#### 2.4.4 Quantification of response amplitude

Amplitude quantification was performed on all face-selective contacts in the same manner for the LF and HF responses. For each reference montage, baseline-corrected amplitudes were computed as the difference between the amplitude at each frequency bin and the average of 48 corresponding surrounding bins (up to 25 bins on each side, i.e., 50 bins, excluding the 2 bins directly adjacent to the bin of interest, i.e., 48 bins). Face-selective responses were quantified separately for each reference montage as the sum of the baseline-subtracted amplitudes at the face frequency from the 1^st^until the 14^th^harmonic (i.e., 1.2 until 16.8 Hz, excluding 6 and 12 Hz) (Hagen et al., 2020,^2021^;Jacques et al., 2022;Jonas et al., 2016).

### 2.5 Contact labeling in the individual anatomy

The exact position of each contact in the individual anatomy was determined by fusing the postoperative CT scan with a T1-weighted MRI. Contacts inside the gray matter were anatomically labeled in the individual anatomy using the same topographic VOTC parcellation as inLochy et al. (2018)based on anatomical landmarks. Contacts located in the white matter were labeled as WM independent of whether the reference was in the white or gray matter. For BIP and LAP, contacts were labeled independently of the location of the neighboring reference contacts (medial for BIP and the two surrounding for LAP). For example, a contact located in the lateral fusiform gyrus (latFG) and referenced to a neighboring contact in the WM would be labeled as latFG.

### 2.6 Removed contacts

Contacts located in brain lesions (or with their local reference in the lesion) visible on structural MRI were excluded from any analyses across all references (*N*= 13). Moreover, for comparing contact distributions of different reference montages, we removed the most medial and most lateral contacts along each electrode for all reference montages, since they could only serve as reference contacts, not active contacts, for LAP. Thus, to compare the spatial distribution of significant contacts for SCA, BIP, LAP, CAR, and REF0, we removed the most medial and most lateral contacts along each electrode in all reference montages (*N*= 802).

### 2.7 Proportion and amplitude maps in Talairach space

In a separate analysis, anatomical MRIs were spatially normalized to determine Talairach coordinates of intracerebral contacts. The cortical surface used to display group maps was obtained from segmentation of the Colin27 brain from AFNI (Cox, 1996) which is aligned to the Talairach space. Using Talairach coordinates, we computed the local proportion and amplitudes of the face-selective intracerebral contacts across the VOTC. Local proportion and amplitudes of contacts were computed in volumes (i.e., “voxels”) of size 12 × 12 × 200 mm (for the X, left–right; Y, posterior–anterior; and Z, inferior–superior dimensions, respectively) by steps of 3 × 3 × 200 mm over the whole VOTC. A relatively large voxel size in the Z dimension was used to collapse across contacts along the inferior–superior dimension. For each voxel, we extracted the following information across all participants in our sample: (i) the number of recorded contacts located within the voxel, (ii) the number of contacts showing a significant response, and (iii) the mean amplitudes in the significant contacts. For each voxel and each reference montage (i.e., SCA, BIP, LAP, CAR, REF0), we computed the proportion of significant contacts over recorded contacts (proportions are critical here since sampling differs across regions), as well as the mean amplitudes over/in the significant contacts. To ensure reliability and reproducibility, we only considered voxels in which at least two participants showed significant responses. For the analysis contrasting the maps of the reference montages (see results below), for each voxel, we determined whether the proportion of significant contacts relative to non-significant contacts was statistically different from zero using a bootstrap procedure in the following way: (i) sampling contacts from the voxel (the same number as the number of recorded contacts in the voxel) with replacement, (ii) determining the proportion of significant contacts for this bootstrap sample and storing this value, (iii) repeating steps (i) and (ii) 5,000 times to generate a distribution of bootstrap proportions and to estimate the*p*-value as the fraction of bootstrap proportions equal to zero.

## 3 Results

### 3.1 Spatial distribution of face-selective activity

**Fig. 2.**
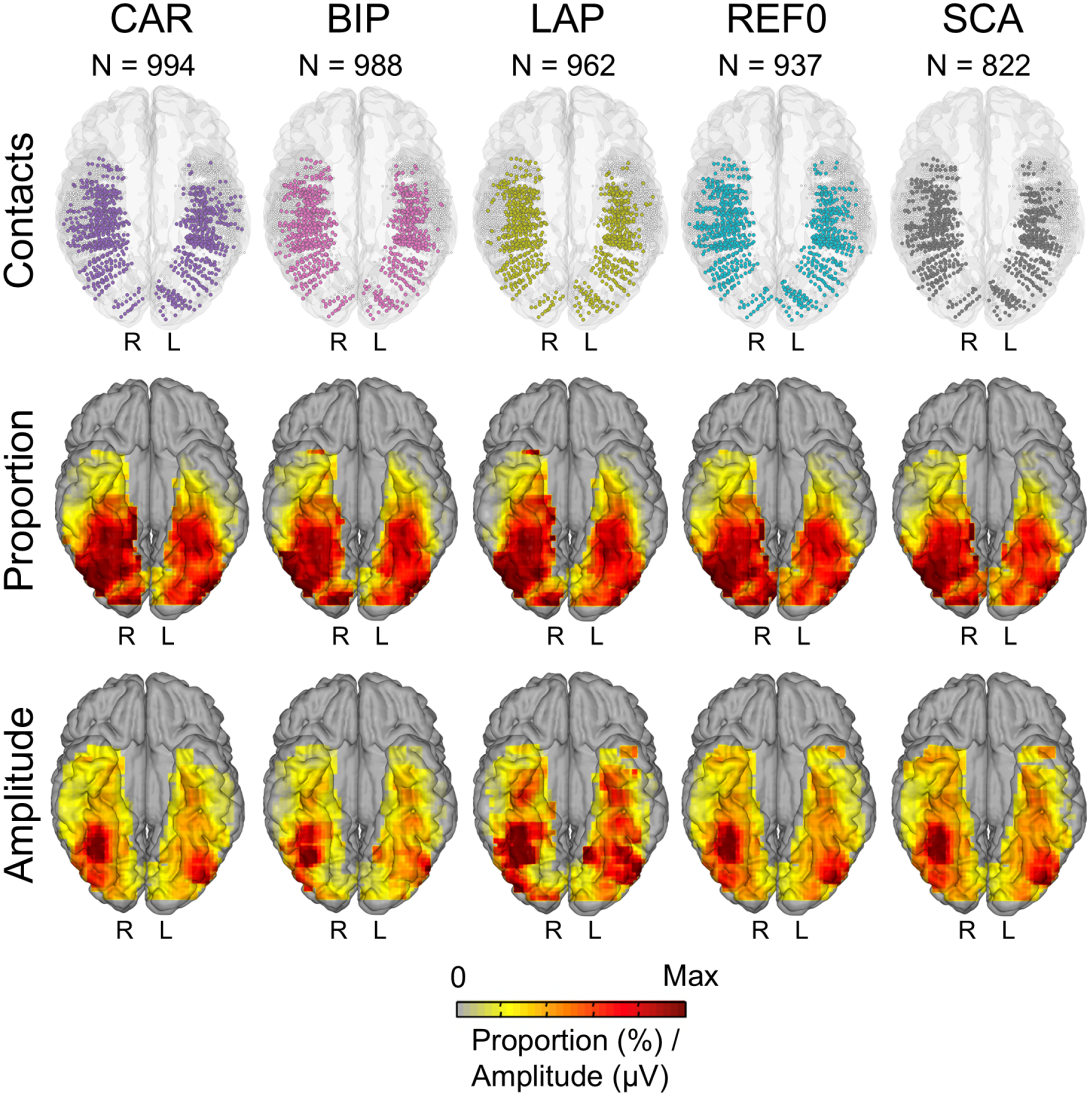
Group maps for low-frequency data. Top: maps of all 3,470 VOTC recording contacts across the 77 individual brains displayed in the Talairach space using a reconstructed cortical surface of the Colin27 brain. Each circle represents a single contact. Color-filled circles correspond to selective contacts. Light-gray-filled circles correspond to contacts on which no selective responses were recorded. Middle and bottom: Proportion and amplitude maps. Local proportions and amplitudes were computed in 12 × 12 mm voxels (for X- and Y-dimensions, respectively) using contacts collapsed over the Z dimension (inferior–superior) for better visualization. For the sake of replicability, only voxels containing significant responses from at least two individual brains were considered. The letters on each hemisphere (R, L) refer to the hemispheric side. CAR, common average reference; BIP, Bipolar; LAP, Laplacian; REF0, zero reference; SCA, scalp.

For the low-frequency data, out of the 3,470 contacts implanted in the gray matter of the VOTC in 77 individual brains, the SCA, REF0, CAR, LAP, and BIP references yielded 822, 937, 994, 962, and 988 significant face-selective contacts, respectively (statistics inSupplementary Table S2andFig. 2, top). To visualize the spatial distribution of the face-selective responses across reference montages, all significant contacts were displayed in the Talairach space for each reference montage, and local proportions (significant contacts/total recorded contacts) were computed at a group level and projected on the cortical surface (Fig. 2, middle). All reference montages yielded bilateral face-selective responses distributed along the entire VOTC. The peak local proportions were highly consistent across references, with most face-selective contacts clustering in the IOG, and along the FG into the ATL, and there was a substantial overlap between all references at the contact level (Supplementary Table S3). The mean X, Y, and Z contact positions within right latFG and right IOG were also consistent across references (Supplementary Table S4), and with previous iEEG (e.g.,Hagen et al., 2020;Jonas et al., 2016) and fMRI studies with large participant samples (Rossion et al., 2012).

**Fig. 3.**
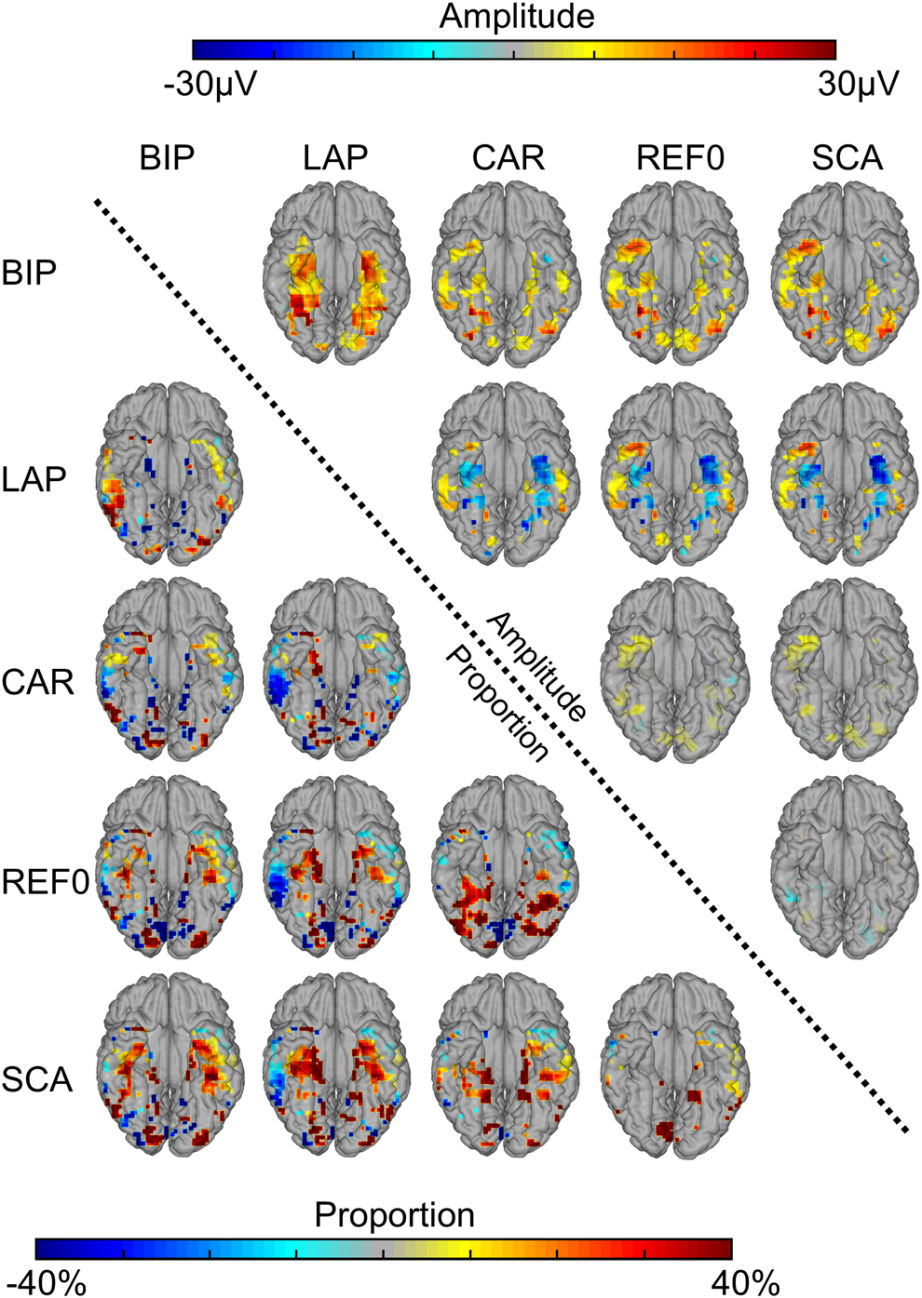
Group contrast maps for low-frequency data. Proportion and amplitude maps contrasting each reference montage: data to the right of the diagonal are amplitude, data to the left of the diagonal are proportion (of significant contacts). Data indicated by row labels are subtracted from the data indicated by the column labels. For example, for amplitudes, row1, column2 = LAP – BIP, and for proportion, row2, column1 = BIP – LAP. Similar plotting convention as proportion and amplitude maps inFigure 2, except that only significant responses are plotted (*p*< 0.05).

Direct comparisons of the proportion maps showed that, relative to all other references, LAP and BIP reduced the proportion of face-selective contacts in posterior- and anterior-lateral regions, while increasing contacts in FG and in the ATL (Fig. 3). A similar pattern was found for CAR compared with SCA and REF0. SCA reduced the proportion of significant contacts in the ATL relative to all other references except REF0. Overall similar patterns were observed in the amplitude maps of the significant contacts (Fig. 2, bottom, 3). Thus, while the different references yielded qualitatively similar face-selective maps, quantitative differences were observed along the medio-lateral and posterior-anterior axis.

All reference montages showed a higher face-selective response amplitude in the right latFG compared with the left (descriptives and statistics inSupplementary Table S5), as initially reported using FPVS-iEEG with an SCA reference (Jonas et al., 2016). However, BIP yielded a weaker right lateralization than SCA, CAR, and REF0 (all*p*< 0.038) but not LAP (*p*= 0.115; two-tailed permutation tests). Similar patterns were observed when computing the lateralization (R – L) as a proportion of total amplitude (R + L), except for LAP and BIP showing numerically smaller lateralization than the other references.

**Fig. 4.**
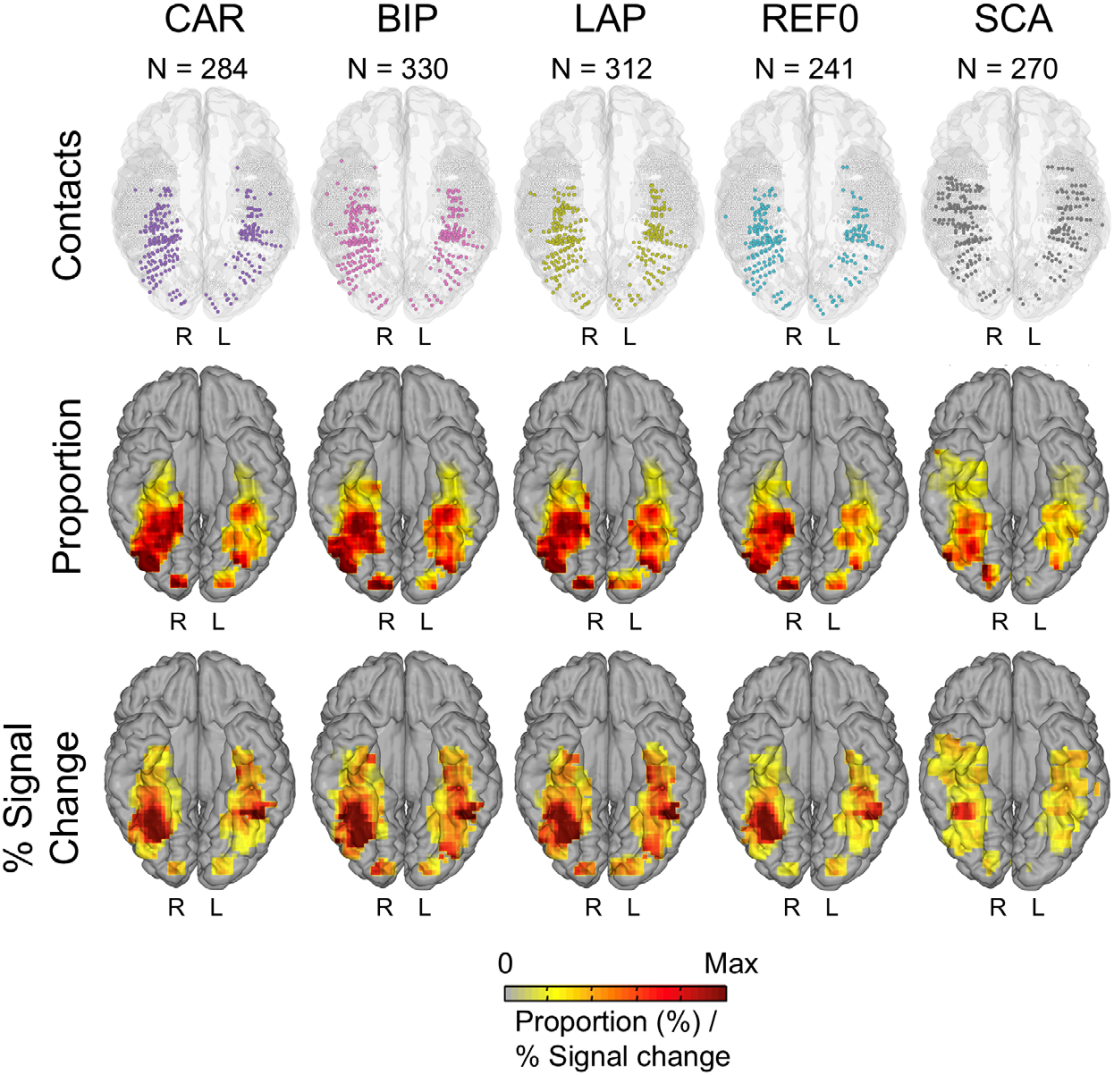
Group maps for high-frequency data. Top: maps of all 3,470 VOTC recording contacts across the 77 individual brains displayed in the Talairach space using a reconstructed cortical surface of the Colin27 brain. Each circle represents a single contact. Color-filled circles correspond to selective contacts. Light-gray-filled circles correspond to contacts on which no selective responses were recorded. Middle and bottom: Proportion and percent signal change maps. Local proportions and percent signal change were computed in 12 × 12 mm voxels (for X- and Y-dimensions, respectively) using contacts collapsed over the Z dimension (inferior–superior) for better visualization. For the sake of replicability, only voxels containing significant responses from at least two individual brains were considered. The letters on each hemisphere (R, L) refer to the hemispheric side. CAR, common average reference; BIP, Bipolar; LAP, Laplacian; REF0, zero reference; SCA, scalp.

For the high-frequency data (between 40 and 160 Hz), SCA, REF0, CAR, LAP, and BIP yielded 270, 241, 284, 312, and 330 significant face-selective contacts, respectively (Fig. 4, top;Supplementary Table S6). For all references, the face-selective contacts clustered in bilateral IOG, and along the bilateral FG (Fig. 4, middle). The mean X, Y, and Z contact positions within the right latFG and right IOG were also consistent across references (Supplementary Table S7). However, for SCA, the peaks were less emphasized, the distribution in the ATL was more widespread (and less restricted to the antFG) (Figs. 4and5), and the spatial overlap with the other references was relatively low (Supplementary Table S8). Similar overall patterns were observed in terms of amplitudes in the significant contacts (Fig. 4, bottom), except that each reference showed trends for a right hemispheric lateralization in the latFG (Supplementary Table S9).

**Fig. 5.**
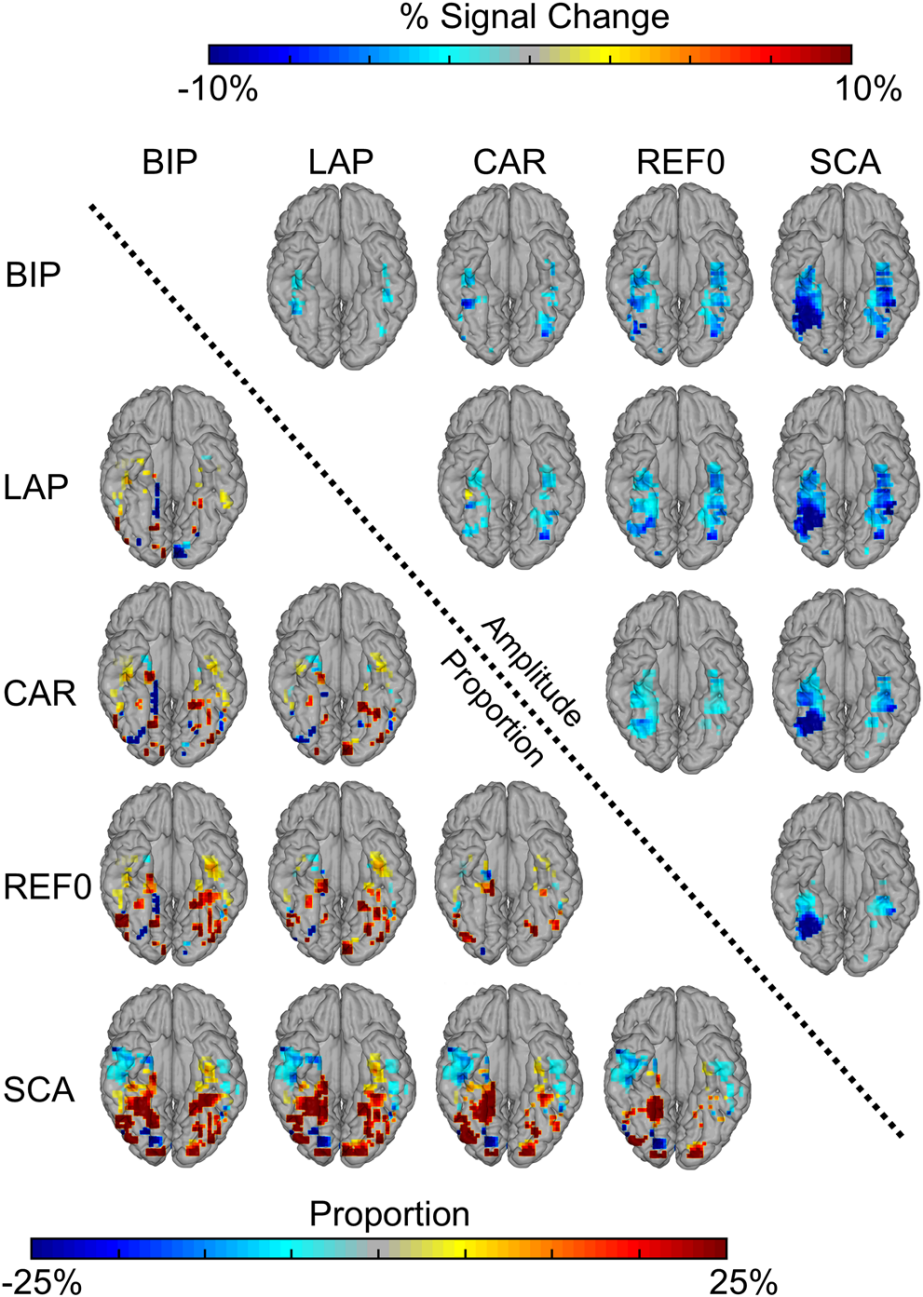
Group contrast maps for high-frequency data. Proportion and percent signal change maps contrasting each reference montage: data to the right of the diagonal are percent signal change, data to the left of the diagonal are proportion (of significant contacts). Data indicated by row labels are subtracted from the data indicated by the column labels. For example, for amplitudes, row1, column2 = LAP – BIP, and for proportion, row2, column1 = BIP – LAP. Similar plotting convention as proportion and percent signal change maps inFigure 4, except that only significant responses are plotted (*p*< 0.05).

### 3.2 The influence of reference montage on strong versus weak face-selective activity

**Fig. 6.**
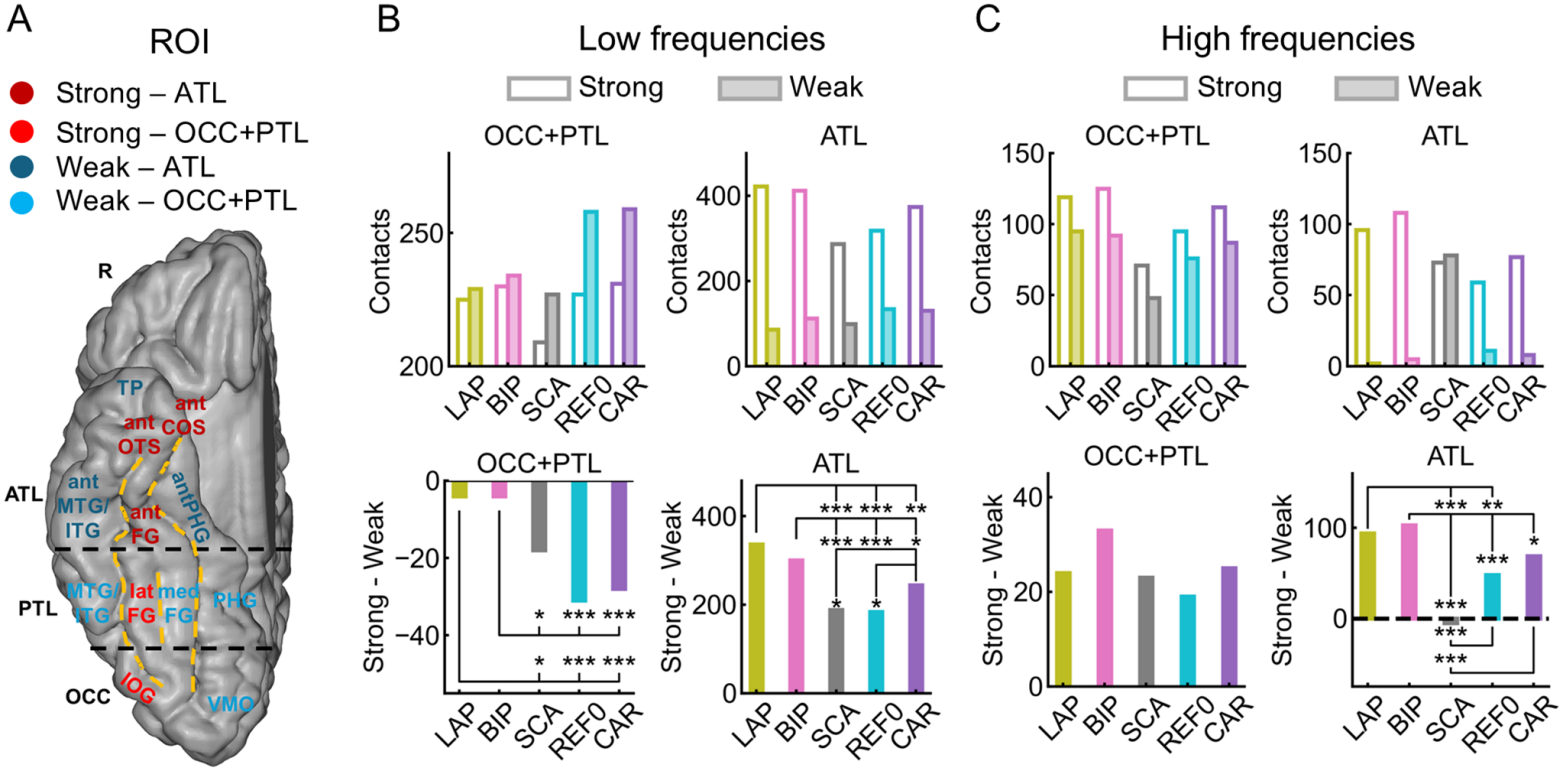
(A) Anatomical regions included in the strong- and weak-selectivity ROIs. In the OCC+PTL, the strong-selectivity ROI includes latFG and IOG, while the weak-selectivity ROI includes VMO, PHG, medFG, and MTG/ITG. In the ATL, the strong-selectivity ROI includes antFG, antCOS, and antOTS, while the weak-selectivity ROI includes antPHG, antMTG/ITG, and TP. Note that only comparisons between references are valid since comparisons within each reference (strong- vs. weak-selectivity ROIs) have unequal sampling and cortical size. Acronyms: OCC, occipital lobe; PTL, posterior temporal lobe; ATL, anterior temporal lobe; IOG, inferior occipital gyrus; VMO, ventromedial occipital; MTG/ITG, middle temporal gyrus/inferior temporal gyrus; FG, fusiform gyrus; PHG, parahippocampal gyrus; OTS, occipito-temporal sulcus; COS, collateral sulcus; ant, anterior; lat, lateral; med, medial. (B, upper) Low-frequency data: significant contact count as a function of ROI and reference montage. (B, lower) Low-frequency data: difference between significant contact count in the strong- and weak-selectivity ROIs (strong–weak). A difference in the positive direction indicates relatively more contacts in the strong- than in the weak-selectivity ROI. (C, upper) High-frequency data: same plotting convention as panel B (upper). (C, lower) High-frequency data: same plotting convention as panel B (lower). *, **, *** represent significance at*p*< 0.05, 0.01, 0.001, respectively.

We examined whether the references differentially influenced the activity in anatomical regions with overall strong versus weak face-selective amplitudes (anatomical regions comprising the “strong” and “weak” ROIs shown inFigure 6A; the split in strong vs. weak face selectivity is consistent withHagen et al., 2020,^2021^;Jacques et al., 2022;Jonas et al., 2016, and current data). Note that only comparisons between references are valid since comparisons within each reference (strong- vs. weak-selectivity ROIs) have unequal sampling and cortical size (Fig. 6A). Separately for the occipital lobe + posterior temporal lobe (OCC + PTL) and the anterior temporal lobe (ATL), we subtracted the number of significant contacts in the strong-selectivity ROI from the number of significant contacts in the weak-selectivity ROI, and directly compared the difference score of the different references (Fig. 6B). In OCC + PTL, BIP and LAP increased the number of contacts relative to the other references in the strong-selectivity ROI relative to the weak-selectivity ROI (Fig. 6B, upper; all*p*< 0.013). In ATL, BIP, LAP, and CAR increased contacts relative to SCA in the strong-selectivity ROI relative to the weak-selectivity ROI (all*p*< 0.028). Moreover, BIP, LAP, and CAR increased contacts relative to REF0 (all*p*< 0.018). Finally, BIP and LAP increased the number of contacts more than CAR in the strong-selectivity ROI than the weak-selectivity ROI (all*p*< 0.05) (Fig. 6B, lower). The same general pattern was observed for the amplitude data (Supplementary Fig. S1B).

For the high-frequency data, in the OCC + PTL, there was no difference across references in terms of the relative contact count in the strong- versus weak-selectivity ROIs (Fig. 6C, bottom; all*p*> 0.112). In contrast, in the ATL, all references had more contacts in the strong- than in the weak-selectivity ROI compared with SCA (all*p*< 0.001). The same pattern was observed for BIP relative to REF0 and CAR (all*p*< 0.034), and LAP relative to REF0 (*p*= 0.002). Qualitatively similar patterns were observed in the percent signal changes within the significant contacts (Supplementary Fig. S1C), except that in OCC + PTL, SCA had a generally low percent signal change in both the strong- and weak-selectivity ROI. Overall, SCA behaved as an outlier compared with BIP, LAP, CAR, and REF0, essentially increasing face selectivity in weak face-selectivity regions in the ATL.

### 3.3 The spread of face-selective amplitudes within recording electrodes

**Fig. 7.**
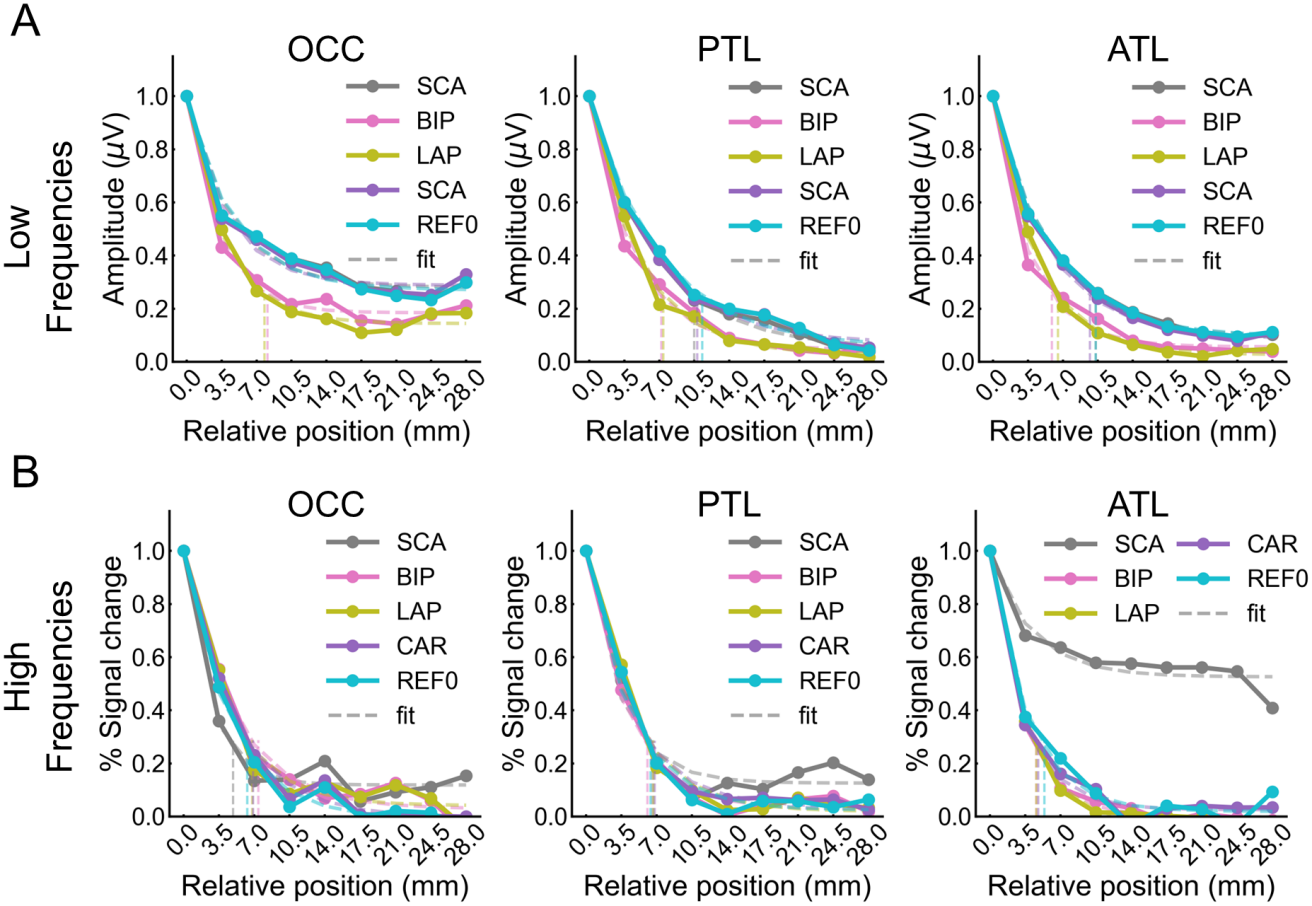
(A) Low-frequency data: Mean variation of face-selective response amplitude as a function of the distance (mm) from the peak amplitude (located at 0 mm), ROI (OCC, PTL, ATL), and reference montage (SCA, BIP, LAP, CAR, REF0). Mean amplitudes have been normalized relative to maximal value (max at 0 mm equals 1) for display only; all analyses were performed on non-normalized data. The spatial extent was estimated for each signal and main region by fitting an exponential decay function to the mean amplitude profile (thin dashed lines) and finding the distance at which the function reached 75% of its amplitude range (vertical dashed lines; an absence of a vertical line indicates that the reference did not reach 75% reduction). To ensure sufficient data, the fitting was restricted to the range from 0 to 28 mm. (B) High-frequency data: same plotting convention as in panel A. To ensure sufficient data, the fitting was restricted to the range from 0 to 21 mm.

We examined the spread of activity around the maximally responsive contact within each electrode shaft. References with better spatial resolution should have a steeper activity decrease away from the maximum contact. For the low-frequency data, across three large posterior–anterior ROIs (OCC, PTL, ATL), all references showed some spatial clustering around the contact with maximum face selectivity, with the second and/or third largest responses next to the maximum and significantly above the amplitude expected if the contacts position was at chance. However, a 75% reduction of the amplitude occurred closer to the maximum contact for local references as compared with the other references in the OCC where SCA, CAR, and REF0 did not reach 75% (Fig. 7A), the PTL (all*p*< 0.006), and the ATL (all*p*< 0.001) (descriptives inSupplementary Table S10). Moreover, this reduction was also larger for BIP than for LAP in the ATL (*p*= 0.041). All other comparisons were not significant (all*p*> 0.226). Thus, while all references showed a spatial clustering of the high face-selective activity (within electrodes), local references reduced the extent of the clustering, suggesting that they sharpen the spatial resolution (descriptives for the number of electrodes with significant contacts inSupplementary Table S11).

For the high-frequency data, in the OCC and PTL, all references had the second largest responses next to the maximum and significantly above the amplitude expected if the contacts position was at chance. In the OCC and the PTL, a 75% reduction occurred at statistically similar positions across all references (Fig. 7B; all*p*> 0.121) (descriptives inSupplementary Table S10). In contrast, in the ATL, SCA did not reach a 75% reduction, while all the other references reached it at statistically similar positions between the 2^nd^and 3^rd^contacts (Fig. 7B; all*p*> 0.194). This suggests that SCA blurs the signals across the ATL electrodes, thereby inflating the number of significant contacts in the ATL (descriptives for the number of electrodes with significant contacts inSupplementary Table S12).

### 3.4 Signal and noise as a function of posterior-to-anterior axis and reference montage

We examined whether the reference montages differed in noise and signal across the posterior–anterior axis of the VOTC. We characterized the Z-scores, signal, and noise along the Y-axis. For each reference and its significant contact, the signal was quantified as the sum of the raw amplitudes (i.e., not baseline corrected) at the face frequency (1^st^until the 4^th^harmonic: 1.2, 2.4, 3.6, and 4.8 Hz), while the noise as the mean of the 25 noise bins on each side of the signal bins, excluding the 2 bins directly adjacent to the bin of interest (i.e., 24 on each side, 48 in total).

**Fig. 8.**
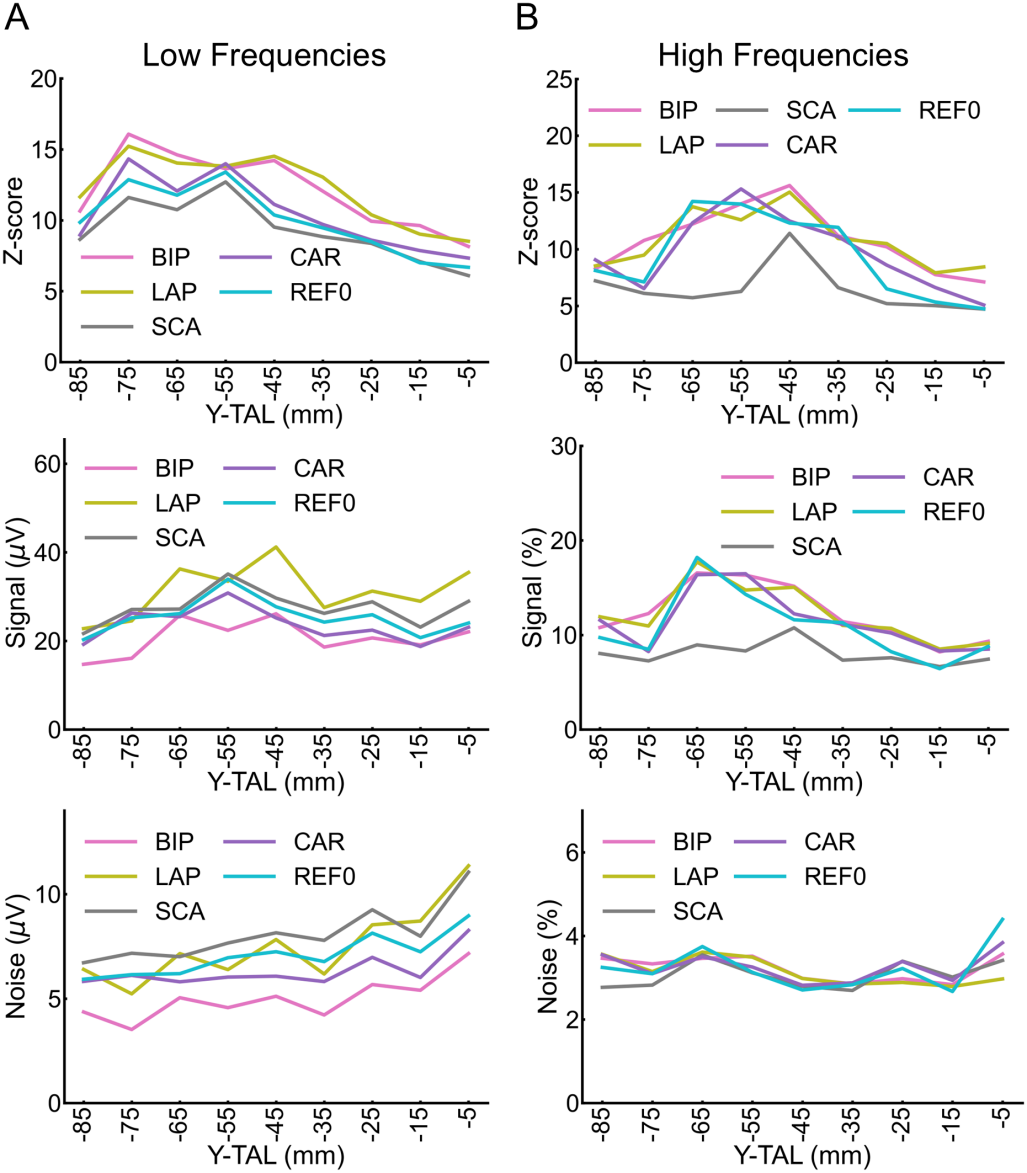
(A) Low-frequency data: Z-score (top), signal amplitude (middle) and noise amplitude (bottom) for Y-Talairach axis (mm) as a function of reference montage (BIP, LAP, SCA, CAR, REF0). Statistical significance for comparisons of different reference montages within each TAL bin is indicated inSupplementary Tables S13–S15. We excluded contacts located below -90 mm and above 0 mm on the Y-Talairach axis due to noise. (B) High-frequency data: same plotting convention as panel B. Statistical significance for comparisons of different reference montages within each Talairach (TAL) bin is indicated inSupplementary Tables S16–S18.

For the low-frequency data, LAP and BIP enhanced Z-scores around -50 to -10 mm on the Y-Talairach axis (posterior–anterior;Fig. 8A, top; statistics inSupplementary Table S13). At points along the Y-Talairach axis, largely corresponding with the change in Z-scores, LAP had higher signal relative to all other reference montages (Fig. 8A, middle), while BIP had lower signal than most reference montages (statistics inSupplementary Table S14). In contrast, BIP was associated with a lower noise level, while LAP had a noise level comparable with the other references (Fig. 8A, bottom; statistics inSupplementary Table S15). Thus, the high Z-scores of LAP were associated mainly with enhanced signal, while for BIP it was associated mainly with reduced noise.

For the high-frequency data, across larger parts of the Y-Talairach axis, SCA had lower Z-scores than the other references, while the remaining references had comparable Z-scores to each other (Fig. 8B, top;Supplementary Table S16). All references had a higher signal than SCA across a large part of the posterior–anterior axis (Fig. 8B, middle; statistics inSupplementary Table S17), while the noise levels were relatively comparably across the references (Fig. 8B, bottom; statistics inSupplementary Table S18). Thus, the generally lower Z-score for SCA was associated with a particularly low signal in high-frequency activity.

### 3.5 Time-courses across reference montages

**Fig. 9.**
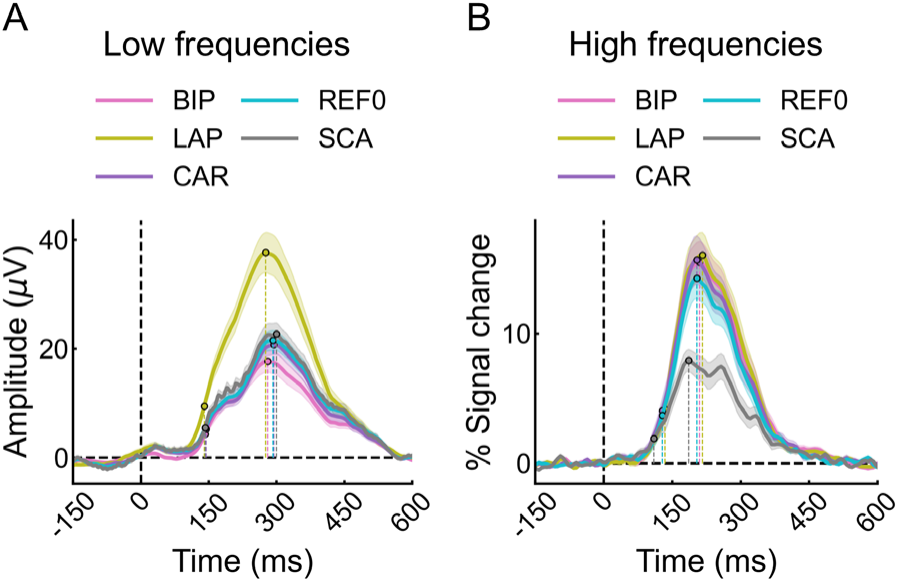
(A) Low-frequency data: Timing of face-selective activity in the bilateral latFG for each reference montage. Shaded area represents 95% confidence intervals (CIs). Dashed lines and dots reflect the time at which the amplitudes reached 25% of peak (“initiation” phase) and peak activity. (B) High-frequency data: same plotting convention as panel (A).

We examined the temporal profile of the face-selective activity in bilateral latFG, a key region for face-selective activity, as well as bilateral ATL, where other analyses revealed differences between the references. For the low-frequency data, overall, each reference’s temporal profiles overlapped, reaching 25% of peak and peak activity at roughly the same times (Fig. 9A;Supplementary Table S19). Similar patterns were observed in the ATL (Supplementary Fig. S2A;Supplementary Table S20). LAP and BIP had overall higher and lower amplitudes, respectively, than the other references, consistent with the analyses in the frequency domain. Thus, the choice of reference montage does not affect the time-course of face-selective activity.

For the high-frequency data, the reference’s temporal profiles in the bilateral latFG also overlapped, reaching 25% of peak and peak activity at roughly the same time (Fig. 9B;Supplementary Table S19). However, SCA had slightly faster onset and peak times than the other references (Supplementary Table S19). All references had higher amplitude than SCA, consistent with the analyses in the frequency domain. Similar patterns were observed in the ATL (Supplementary Fig. S2B;Supplementary Table S20), except that the response for SCA was less sustained, had similar amplitude, and similar onset time as the other references. Thus, the choice of reference montage did not severely influence the onset and peak timing parameters of the face-selective activity, except in some cases for SCA.

### 3.6 Face selectivity in white matter contacts

**Fig. 10.**
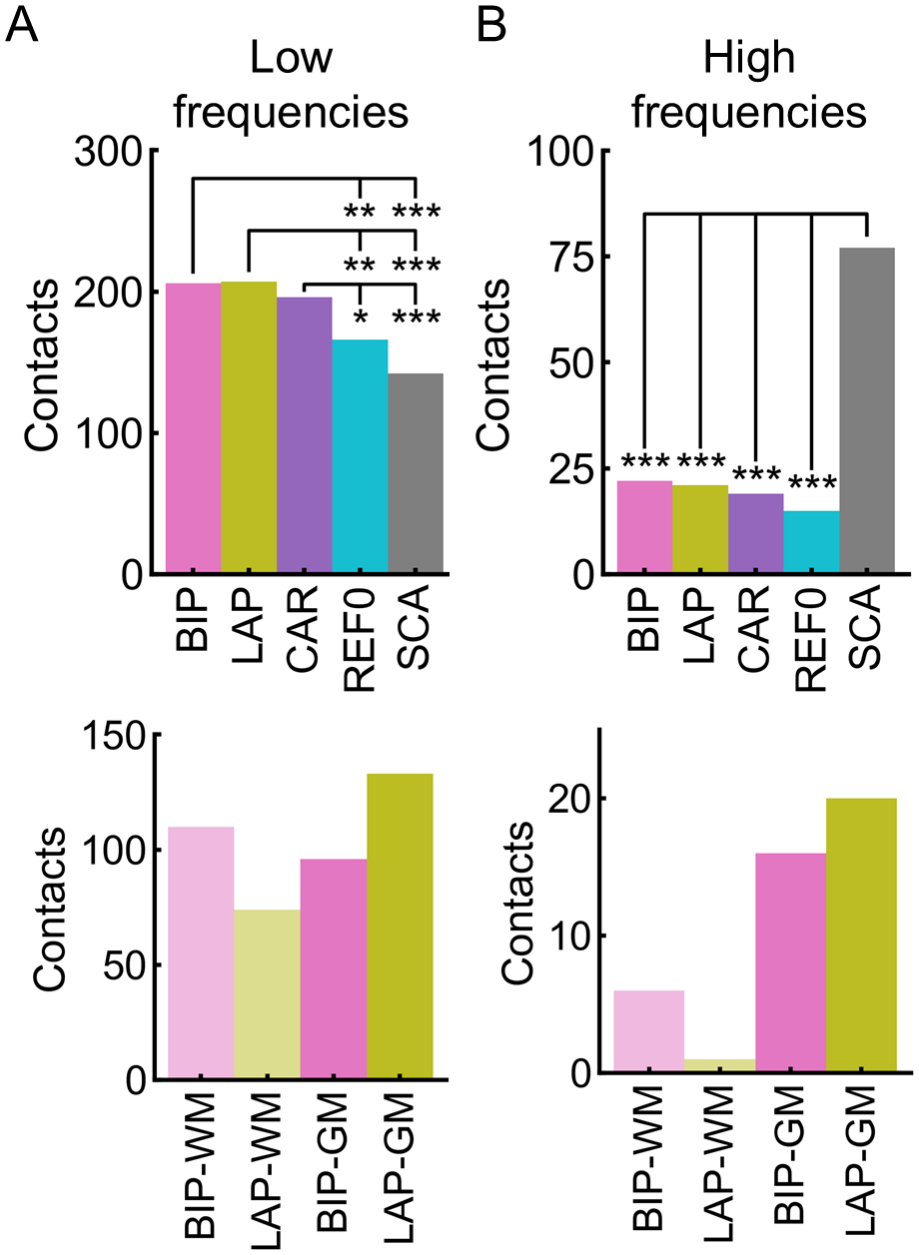
Significant white matter (WM) contacts. (A) Low-frequency data. Top: number of significant WM contacts as a function of reference montage (BIP, LAP, CAR, REF0, SCA). Bottom: number of significant WM contacts as a function of local reference montage (BIP, LAP) and reference position(s) (WM, GM). (B) High-frequency data. Same plotting convention as in panel A. *, **, *** represent significance at*p*< 0.05, 0.01, 0.001, respectively.

Finally, we examined the activity recorded in contacts located in the white matter (WM), with the assumption that they reflect volume-conducted responses from neighboring gray matter (GM). For the low frequencies, there were a substantial number of face-selective contacts in the WM for all reference montages, with LAP, BIP, and CAR having the most WM contacts (Fig. 10A, top; statistics inSupplementary Table S21). However, these differences were less pronounced—or non-existent—when considering that these references had overall more significant contacts in the gray matter also (e.g., due to reduced noise or increased signal [see earlier sections];Supplementary Table S21). Finally, we examined whether WM activity could reflect gray matter activity in the reference contact(s). For LAP and BIP, where the reference contact(s) can be localized, there was a large proportion of contacts with a WM reference (Fig. 10A, bottom; descriptives inSupplementary Table S22), suggesting that at least for BIP and LAP, signal transfer from a GM reference could not fully account for all the significant WM contacts. Overall, this could suggest that both local and global references in low-frequency activity contain a fair amount of volume-conducted signals.

For the high-frequency data, SCA, BIP, LAP, CAR, and REF0 had 77, 22, 21, 19, and 15 significant white matter contacts, respectively (Fig. 10B, top; statistics inSupplementary Table S23). The substantially higher number of WM contacts for SCA remained when considering its overall number of significant contacts in the GM (Supplementary Table S23). This is consistent with previous high-frequency analysis, suggesting that SCA diffuses the high-frequency selective activity to a larger extent than the other references. Finally, for LAP and BIP, most of the WM contacts had the reference in the GM (Fig. 10B, bottom; descriptives inSupplementary Table S24), suggesting that at least for BIP and LAP, the WM activity reflect GM activity that is transferred from the reference channels.

## 4 Discussion

### 4.1 Overall spatial distribution and timing profiles of face selectivity unaffected by reference montage

Overall, the spatial distribution of the face-selective contacts and their amplitudes were largely similar for the five tested references montages, both for low- and high-frequency data, except for SCA in high frequencies. Specifically, all reference montages showed similar face-selective activity in posterior–anterior and medial–lateral regions of the VOTC, with peak activity in and around the posterior and anterior FG. Moreover, all references showed the well-known right hemispheric dominance of face-selective amplitudes in the latFG (Gao et al., 2018;Sergent et al., 1992; seeRossion & Lochy, 2022), albeit with differences in magnitude (see below). Finally, all references yielded rather similar timing profiles of face-selective responses, except for SCA in high frequencies. Thus, with the exception of SCA for high frequencies, the choice of reference montage does not appear to have much influence on the spatial and temporal distribution of face-selective neural activity in the human VOTC.

### 4.2 Advantages of local reference montages

For low frequencies, while we did not observe large spatial differences across montages, we still found subtle local differences. The most important difference was that local references (BIP, LAP) increased face selectivity in typically strong-selectivity regions (i.e., IOG, FG, and adjacent sulci) relative to more lateral and medial regions (weak selectivity), particularly in the ATL (antFG and adjacent sulci antOTS and antCOS). In other words, BIP and LAP emphasized focal peaks of selectivity, in regions that are known to be critical for face recognition, and de-emphasized weaker selectivity, in areas thought to be less important for face recognition. This was mainly related to a reduction of noise level for BIP and to an increase of signal amplitude for LAP. One possible reason for the reduction of face selectivity in lateral and medial regions is reduction of volume-conducted signals, as suggested by the reduction of the spread of activity from the maximum contact along the electrode. These advantages of local references are consistent with previous studies showing that some local references (LAP) enhance task-related activity and minimize inter-channel correlations (Li et al., 2018). For high frequencies, except for SCA that yielded less significant contacts overall, differences were more subtle, being mostly found in the ATL, with higher SNR for local references and more contacts in strong selectivity regions compared with the common references.

Thus, while the choice of reference is unlikely to influence the spatial distribution of responses, it could influence measurements in more subtler ways. Especially, the use of local references could increase measured face selectivity in specific regions such as the ATL, which is especially important given its role in face recognition (e.g.,Jonas et al., 2015;Rossion et al., 2024;Volfart et al., 2022). The SNR in the ATL is lower than posterior regions for both low- and high-frequency activity (Jacques et al., 2022) and local references better capture low-SNR responses, either due to increased signal, reduced noise, or both.

### 4.3 Reference montages increasing face-selective activity in the white matter

It is generally believed that weak local field potentials measured in contacts situated in WM reflect volume-conducted signals from nearby GM. Interestingly, for low-frequency activity, we found that local references substantially increased measured face-selective activity in the WM, despite their assumed role of reducing volume-conducted signals compared with common references (Arnulfo et al., 2015;Mercier et al., 2017,^2022^). For local references, even after excluding WM contacts with a reference in the GM (where significance could reflect the reference activity), there were still a substantial portion of significant WM contacts. This suggests that local references do not fully abolish volume-conducted activity, presumably mainly removing volume conduction from far sources (i.e., in the far field of the dipole). Surprisingly, SCA yielded fewest WM contacts in low frequencies, despite the large distance between the active and the reference electrodes, perhaps because of a higher level of noise. In contrast, for high-frequency responses, the reference type did not influence face-selective responses in the WM, except SCA, which substantially increased the number of significant WM contacts. This could potentially be driven by the weak or absent high-frequency (periodic) electrophysiological signals on the scalp (Gotman & Crone, 2012), artificially increasing unsignificant high-frequency signals in the WM. Alternatively, or in addition, the projection of high-frequency noise picked up by scalp electrodes and periodically related to face stimulation (e.g. micro-saccades;Yuval-Greenberg et al., 2008) into the iEEG signal may produce artificial high-frequency signals detected in the WM (due to the reduced presence of neural activity in these sites). Interestingly, local references in high-frequency signals clearly reduced the proportion of WM contacts with a WM reference (WM-WM) compared with low frequencies, which points to an advantage of using local references for high-frequency signals.

Thus, our results highlight that the issue of choosing reference montage for reducing volume-conducted signals is complex, and likely depends on several factors, including active-reference distance and levels of noise and signal.

### 4.4 Considerations when choosing reference montage

Different reference montages are thought to come with different (dis)advantages (e.g.,Mercier et al., 2022). SCA is thought to capture a large degree of volume-conducted neural activity (Ball et al., 2009;Jerbi et al., 2009;Katz et al., 2020;Kovach et al., 2011;Mercier et al., 2022). Consistent with this, face-selective responses for SCA were found in lateral regions for both frequencies (but especially in the lateral ATL for high-frequency responses), and in the WM for high-frequency responses. Moreover, the SCA reference could be contaminated by artifacts such as external noise (i.e., ambient 50 or 60 Hz), or physiological artifacts (muscle activity, blinks, eye, or head movements). This “reference contamination” could have disproportionately affected recordings in regions with low SNR, such as the ATL. In addition, the ATL is known to be specifically affected by eye saccade/blink artifacts of the SCA reference (Katz et al., 2020;Kovach et al., 2011). While being broadband, these artifacts have more energy in low-frequency bands, which may contribute to finding fewer face-selective contacts in the ATL for SCA compared with other reference montages for low-frequency activity. Interestingly however, since these artifacts are broadband, the frequency-tagging approach used here (with a high-frequency resolution and a narrow frequency band of interest) is relatively immune to such artifacts. For high frequencies, as discussed earlier, the SCA reference acts as an outlier compared with other references and should probably be avoided in most situations.

The CAR reference montage removes common signal across all contacts. Studies have shown that CAR can decrease inter-contact correlations compared with, for example, a WM average reference or GM average reference (Li et al., 2018;Mercier et al., 2017). In addition, our results show that CAR has relatively low noise and a large number of significant contacts, especially in the ATL. However, since it assumes homogeneous spherical sampling, its validity for iEEG recordings, especially with SEEG, can be questioned (Mercier et al., 2022;Parish et al., 2023; for scalp EEG seeDesmedt et al., 1990;Kayser & Tenke, 2010). This violation could result in activity from more densely sampled regions/hemisphere to be artificially projected to contacts over less densely sampled regions. REF0 is related to CAR, by also removing common electrode activity, but using an electrode-weighted average. However, this montage is relatively infrequently used for (S)EEG, and thus lacks validation for task-related functional activity. Here we show that, for low frequencies, it is almost identical to SCA in most parameters that we examined: spatial distribution of proportion and amplitudes, number of contacts in weak and strong-selectivity regions, spatial spread, and response timing.

Local reference montages isolate local activity by removing common signal in adjacent contacts and reduce signal correlation between contacts (Li et al., 2018;Mercier et al., 2017). We found that both BIP and LAP increased face selectivity in strongly selective relative to “weakly” regions, consistent with enhancing local activity. However, these references may be suboptimal for recordings of regions that contain a large portion of contacts at the electrode edge, since they can only serve as reference. For example, in our study, LAP reduced the total active sites (and thus potentially significant contacts) from 4,272 to 3,470 sites (802 contacts or ~19% reduction) compared with the common references (SCA, REF0, CAR). For low-frequency responses, local montages also led to absolute and relative reduction of the well-known right hemispheric dominance for face-related neural activity in the VOTC (Gao et al., 2018;Sergent et al., 1992; seeRossion & Lochy, 2022), perhaps due to signal cancellation in strong face-selective sources (Zaveri et al., 2006), if the cortical extent of the neural source is equal to or larger to inter-contact distance (1.5 mm from edge to edge). Finally, at the contact level, BIP and LAP could have quite variable effects on signal localization depending on dipole orientations, signal amplitude and phase values across contacts (Zaveri et al., 2006).

### 4.5 Generalizability of our findings

An important issue is the degree to which our findings are generalizable to electrophysiological mapping of other cognitive functions, in different brain regions and different recording techniques. For several reasons, we argue that the approach used here provides good generalizability of our results, at least to primary and association cortices. First, we recorded responses from a large sample of individual brains, sampling the wide cortical surface recruited selectively by face stimuli in the human VOTC, from the occipital to the temporal poles, encompassing various types of cortices, architectonics, and cognitive systems (primary and association cortices, unimodal visual, and multimodal semantic cortices). Second, while electrocorticography (ECoG) mainly records activity from the gyral surface and has advantages in sampling size and homogeneity, SEEG samples a wider range of anatomical structures or cortical geometries (i.e., sulci, gyri) and can, therefore, record multiple dipole orientations at various locations and distances to active and reference sites. Note that ECoG may present some specificities compared with SEEG for local references related to (1) the common use of 2D grids instead of 1D electrode linear arrays and (2) the limitation of recording sites to the gyral surface, potentially reducing the sensitivity to distal sulcal activities. Third, the frequency-tagging stimulation approach provides objectivity of response identification and quantification across reference methods. Even though this approach remains relatively rare compared with slow temporally jittered event-related stimulation methods, periodic streams of visual stimuli are a longstanding stimulation method in human electrophysiology (Adrian & Matthews, 1934; intracranial EEG:Kamp et al., 1960). Most importantly, the current approach has been extensively validated in EEG (e.g.,Quek & Rossion, 2017;Retter & Rossion, 2016;Rossion et al., 2015), iEEG (seeRossion et al., 2018,^2023^for review) and other methods (MEG:Hauk et al., 2021, fMRI:Gao et al., 2018), providing the same functional responses as conventional approaches but with significant advantages in sensitivity and objectivity. Fourth, we tested not only responses from the lower range of the frequency spectrum (<30 Hz) but also high-frequency responses (40–160 Hz, or broadband gamma activity), which have become the primary center of interest for an increasing number of human intracranial recording studies over the last 15 years (e.g.,Miller et al., 2007;Nir et al., 2007;Ray et al., 2008).

Since a large portion of iEEG studies on face- and category-selective activity focus on characterizing their temporal characteristics, we also examined the effect of reference montage on face-selective temporal activity, providing empirical evidence that reference montage does not influence these time-course properties. Also, while it is well known that the VOTC contains other category-selective responses, including to human bodies (Peelen & Downing, 2005), written words (e.g.,Devlin et al., 2006), and scenes and buildings (e.g.,Aguirre et al., 1998;Epstein & Kanwisher, 1998), we focused on face-selective activity, which is arguably the strongest, most distributed, and most studied form of category-selective responses in the VOTC. Although face- and category-selective regions show a functional dissociation, they often share some cortical regions, and so there is no reason to believe that our results will not generalize to other category-selective responses in the VOTC.

Finally, the generalizability of our findings to individual brain analyses (i.e., single-subject studies) remains unclear. That is, while the different reference montages had little effect on the group spatial distribution of face-selective activity here, we cannot exclude that they substantially affect spatial distributions of activity in individual brains, depending on the configuration of electrodes and individual cortical geometry. Individual brain analyses were not studied in the present study; thus, future work should examine the effect of referencing at the single-subject level.

## 5 Conclusion

Although the reference problem in electrophysiological recordings is complex, and other existing refence montages, for example, different scalp electrodes, cranial or subdermal electrodes, white matter site, median common average, electrode shaft reference, gray–white matter common reference, independent component analysis, REST, (Li et al., 2018;S. Liu et al., 2021;Y. Liu et al., 2015;Mercier et al., 2022;Michelmann et al., 2018;Parish et al., 2023;Yao, 2001) were not tested here, our findings provide some recommendations for future group iEEG studies. Even more so than for EEG recordings, there may be no optimal reference for iEEG studies (Mercier et al., 2022), with some references being more suited for specific research questions, anatomical regions, types of analyses, and responses frequency range (Dien, 1998). For example, our results indicate that local reference methods increase SNR and may, therefore, be better suited to the recording of low-amplitude responses (here the ATL). If the goal is to study medial (e.g., PHG) or lateral structures (e.g., MTG) in SEEG, we do not recommend an LAP montage that discards contacts in these regions. If the goal is to compare response amplitudes across regions, one should be cautious with local reference methods as they may paradoxically disadvantage regions generating the largest response amplitudes because of signal cancellation in the low-frequency range (e.g., here, the right latFG). For high-frequency responses, our findings indicate that the SCA reference is not recommended. In general, as suggested in EEG research (Katznelson, 1981), we recommend ensuring consistency of the results across reference methods, using at least two reference schemes, preferably one common (e.g., SCA, CAR, REF0, or white matter despite not tested here) and one local method (BIP or LAP), given their different advantages.

## Supplementary Material

Supplementary Material

## Data Availability

The data and code used for this project are available upon request.
