# Supplementary material for: Intracranial EEG referencing for large-scale category-selective mapping in the human ventral occipito-temporal cortex
Source: Imaging Neurosci (Camb). 2025 Feb 24;3:imag_a_00479. doi: 10.1162/imag_a_00479 (PMC12319787; doi:10.1162/imag_a_00479)
Supplement: Supplementary Material [file imag_a_00479-supp.pdf]

SI for *Intracranial EEG referencing for large-scale category-selective mapping in the human ventral occipito-temporal cortex*

	**Authors**	**Reference montage**
1	Allison et al.1994a	Mastoid
2	Allison et al. 1994b	Mastoid
3	Allison et al. 1999	Mastoid
4	Allison et al. 2002	Mastoid
5	Bansal et al. 2012	Not reported
6	Agam et al. 2010	Not reported
7	Barbeau et al. 2008	SCA (Fz)
8	Boring et al. 2021	Not reported
9	Boring et al. 2024	Not reported
10	Chong et al. 2013	Vertex
11	Davidesco et al. 2014	CAR
12	Engell et al. 2010	Mastoid
13	Engell et al. 2011	Mastoid or skull electrode
14	Engell et al. 2014a	Mastoid or skull electrode
15	Engell et al. 2014b	Skull electrode
16	Fisch et al. 2009	Extracranial electrode
17	Ghuman et al. 2014	Not reported
18	Grossman et al. 2019	CAR
19	Hagen et al. 2020	SCA (FPz) or WM
20	Hagen et al 2021	SCA (FPz) or WM
21	Halgren et al. 1994a	Not reported
22	Halgren et al. 1994b	Not reported
23	Hamamé et al. 2014	BIP
24	Jacques et al., 2020	SCA (FPz) or WM
25	Jacques et al., 2022	BIP
26	Jacques et al. 2016	CAR (subset of contacts)
27	Jonas et al. 2016	SCA (FPz) or WM
28	Kadipasaoglu et al. 2016	CAR
29	Kadipasaoglu et al. 2017	CAR
30	Keller et al. 2017	CAR
31	Klopp et al. 2000	Nose tip
32	Kuzovkin et al. 2020	BIP
33	Lachaux et al. 2005	BIP
34	Li et al. 2019	Subdural
35	Liu et al. 2009	Not reported
36	Matsuo et al. 2013	SCA or CAR
37	McCarthy et al. 1999	Mastoid

38	Miller et al. 2017	CAR
39	Miller et al. 2016	CAR
40	Mundel et al. 2003	Not reported
41	Murphey et al. 2009	Not reported
42	Nobre et al. 1994	Not reported
43	Norman et al. 2019	BIP (WM for HIP contacts)
44	Parvizi et al. 2012	CAR (subset of contacts)
45	Privman et al. 2011	CAR
46	Privman et al. 2007	CAR
47	Puce et al. 1997	Not reported
48	Puce et al. 1999	Mastoid
49	Rangarajan et al. 2014	CAR
50	Rangarajan et al. 2020	CAR
51	Rangarajan, Parvizi, 2015	CAR
52	Rosburg et al. 2010	Mastoids
53	Sanada et al. 2021	CAR
54	Sato et al. 2014	Single or avg subdural
55	Schalk et al. 2017	CAR
56	Schrouff et al. 2020	CAR
57	Schwartz et al. 2023	Subdural
58	Seeck et al. 1997	Not reported
59	Tanji et al. 2012	WM
60	Vidal et al. 2010	Not reported

Table S1. *Summary of reference montages used by 60 studies on face-related iEEG activity, recorded from 1994 to 2024.*

	N	%	CAR	BIP	LAP	REF0	SCA
CAR	994 (69)	29%	NA	.03, .873	.7, .393	2.3, .127	22.1, < .001
BIP	988 (71)	28%	.03, .873	NA	.5, .490	1.9, .172	20.6, < .001
LAP	962 (72)	28%	.7, .393	.5, .490	NA	.5, .50	14.8, < .001
REF0	937 (71)	27%	2.3, .127	1.9, .172	.5, .500	NA	10.1, .002
SCA	822 (69)	24%	22.1, < .001	20.6, <.001	14.8, < .001	10.1, .002	NA

Table S2. *Grey matter contact descriptives (columns 2 - 4) and statistics (columns 5 - 9) as a function of reference montage (rows). Values in brackets of 2nd column indicate number of significant subjects. Values in column 3 indicate the amount in percentage relative to the total number of implanted contacts (3470 contacts implanted in the gray matter across 77 unique brains) (percentages rounded to zero decimals). Statistical comparisons report the t and p-values, respectively, from independent t-tests (t values rounded to one decimal).*

	LAP	BIP	REF0	CAR	SCA
**LAP**	-	78%	72%	73%	76%
**BIP**	81%	-	72%	72%	76%
**REF0**	70%	69%	-	73%	97%
**CAR**	75%	72%	77%	-	91%
**SCA**	65%	63%	85%	75%	-

Table S3. *Low frequency data. Percentage of contacts that are shared across references. Computed as the number of shared contacts divided by the total number of contacts in each reference. For example, the value in row1, column6 reflects % of SCA contacts also significant in LAP, and is computed by the total number of contacts shared between SCA and LAP divided by the total number of SCA contacts.*

Anatomical region	Reference montage	X (mm)	Y (mm)	Z (mm)
Right latFG	SCA	38.8	-46.56	-15.07
Right latFG	BIP	39.75	-46.02	-15.23
Right latFG	LAP	38.69	-46.3	-15.16
Right latFG	CAR	39.3	-46.04	-14.98
Right latFG	REF0	39.08	-46.24	-14.95
Right IOG	SCA	41.31	-68.02	-8.45
Right IOG	BIP	41.82	-68.1	-8.81
Right IOG	LAP	41.91	-67.73	-9.0
Right IOG	CAR	41.43	-68.17	-8.57
Right IOG	REF0	41.54	-68.08	-8.61

Table S4. *Low frequency data. Mean X, Y, and Z Talairach coordinates for significant contacts as a function of ROI (right latFG, right IOG) and reference montage (SCA, BIP, LAP, CAR, REF0).*

Reference montage	Right hemisphere	Left hemisphere	Difference (right – left)	Statistics	Proportion (R-L / R+L)
**SCA**	48.23 µV	21.96 µV	26.27 µV	$t(119) = 4.69, p < .001$	.37 µV
**BIP**	37.42 µV	22.62 µV	14.8 µV	$t(139) = 2.29, p = .024$	.25 µV
**LAP**	60.71 µV	34.94 µV	25.77 µV	$t(132) = 2.32, p = .022$	.27 µV
**REF0**	45.82 µV	20.25 µV	25.57 µV	$t(132) = 4.78, p < .001$	.39 µV
**CAR**	44.26 µV	19.05 µV	25.21 µV	$t(138) = 4.9, p < .001$	.40 µV

Table S5. *Low frequency data. Face-selective amplitude in right and left lateral fusiform gyrus. The statistics use an independent t-test for equal variance if the variance ratio is less than four or unequal variance if the variance ratio is larger than four. BIP and LAP had equal variance, while SCA, REF0, and CAR had unequal variance.*

	N	%	SCA	BIP	LAP	REF0	CAR
SCA	270 (45)	8%	NA	6.6, .010	3.3, .069	1.8, .183	.4, .535
BIP	330 (64)	10%	6.6, .010	NA	.6, .456	15.1, < .001	3.8, .052
LAP	312 (60)	9%	3.3, .069	.6, .456	NA	9.9, .002	1.4, .230
REF0	241 (55)	7%	1.8, .183	15.1, < .001	9.9, .002	NA	3.8, .051
CAR	284 (59)	8%	.4, .535	3.8, .052	1.4, .230	3.8, .051	NA

Table S6. *High frequency data. Grey matter contact descriptives (columns 2 - 4) and statistics (columns 5 - 9) as a function of reference montage. Values in brackets of 2nd column indicate number of significant subjects. Values in column 3 indicate the amount in percentage relative to the total number of implanted contacts (3470 contacts implanted in the gray matter across 77 unique brains) (percentages rounded to zero decimals). Statistical comparisons report the t and p-values, respectively, from independent t-tests (t values rounded to one decimal).*

Anatomical region	Reference montage	X (mm)	Y (mm)	Z (mm)
Right latFG	SCA	38.14	-45.60	-15.95
Right latFG	BIP	38.41	-46.57	-15.65
Right latFG	LAP	38.38	-45.89	-15.85
Right latFG	CAR	37.93	-46.29	-15.65
Right latFG	REF0	38.14	-45.76	-15.67
Right IOG	SCA	39.70	-66.56	-8.28
Right IOG	BIP	42.22	-67.70	-8.36
Right IOG	LAP	41.22	-67.84	-8.52
Right IOG	CAR	40.97	-68.37	-8.56
Right IOG	REF0	40.86	-68.21	-8.39

***Table S7**. High frequency data. Mean X, Y, and Z Talairach coordinates for significant contacts as a function of ROI (right latFG, right IOG) and reference montage (SCA, BIP, LAP, CAR, REF0).*

	LAP	BIP	REF0	CAR	SCA
**LAP**	-	74%	77%	75%	42%
**BIP**	79%	-	73%	73%	42%
**REF0**	59%	53%	-	77%	47%
**CAR**	68%	62%	91%	-	46%
**SCA**	36%	34%	52%	44%	-

***Table S8.** High frequency data. Percentage of contacts that are shared across references. Computed as the number of shared contacts divided by the total number of contacts in a given reference. For example, the row1, column6 reflect % of SCA contacts also significant in LAP, and is computed by the total number of contacts shared between SCA and LAP divided by the total number of SCA contacts.*

Reference montage	Right hemisphere	Left hemisphere	Difference (R – L)	Statistics	Proportion (R-L / R+L)
**SCA**	.07 %	.04 %	.03 %	*t*(47) = 1.97, *p* = .057	.27 %
**BIP**	.13%	.08 %	.05 %	*t*(77) = 1.93, *p* = .057	.24 %
**LAP**	.14 %	.08 %	.06 %	*t*(71) = 1.94, *p* = .056	.27 %
**REF0**	.11 %	.07 %	.04 %	*t*(58) = 1.40, *p* = .168	.22 %
**CAR**	.13 %	.08 %	.05 %	*t*(63) = 1.51, *p* = .136	.24 %

***Table S9.** High frequency data. Face-selective amplitude in right and left lateral fusiform gyrus. The statistics is using an independent t-test for equal variance if the variance ratio is less than four or unequal variance if the variance ratio is larger than four. Only SCA had unequal variance.*

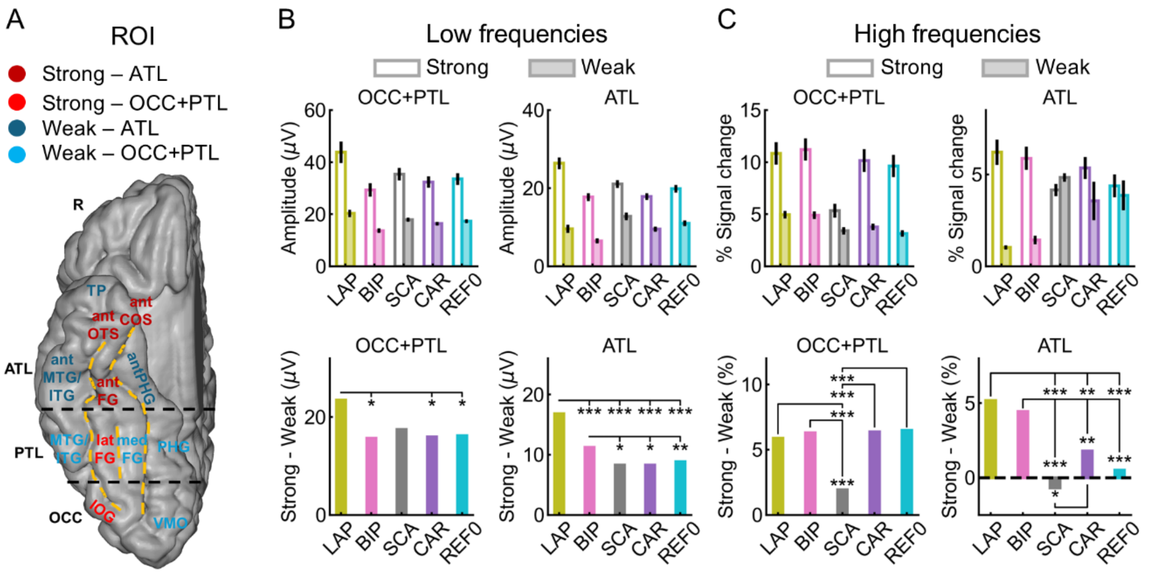

Figure S1. A. *Anatomical regions included in the strong- and weak-selectivity ROIs. In the OCC+PTL, the strong-selectivity ROI includes latFG and IOG, while the weak-selectivity ROI includes VMO, PHG, medFG, and MTG/ITG. In the ATL, the strong-selectivity ROI includes antFG, antCOS, and antOTS, while the weak-selectivity ROI includes antPHG, antMTG/ITG, and TP****.*** *Note that only comparisons between references are valid since comparisons within each reference (strong- vs. weak-selectivity ROIs) have unequal sampling and cortical size.* ***B, upper.*** *Low frequency data: face-selective amplitudes in significant contacts as a function of ROI and reference montage.* ***B, lower.*** *Low frequency data: difference between the face-selective activity in the strong- and weak-selectivity ROIs (strong - weak). A difference in the positive direction indicates relatively higher selective amplitudes in the strong- than the weak-selectivity ROI.* ***C, upper.*** *High frequency data: same plotting convention as panel B (upper) for the percent signal change.* ***C, lower.*** *High frequency data: same plotting convention as panel B (lower) for the percent signal change. Error bars represent SEM. *, **, *** represent significance at p < .05, .01, .001, respectively.*

Reference	Low frequency data			High frequency data		
	OCC	PTL	ATL	OCC	PTL	ATL
SCA	> 28 mm	10.5 mm	10.3 mm	4.9 mm	6.6 mm	> 28 mm
BIP	8.1 mm	7.2 mm	5.9 mm	7.4 mm	6.1 mm	4.6 mm
LAP	7.8 mm	7.4 mm	6.5 mm	6.8 mm	6.7 mm	4.6 mm
CAR	> 28 mm	11.3 mm	9.7 mm	6.9 mm	6.8 mm	4.8 mm
REF0	> 28 mm	10.8 mm	10.3 mm	6.3 mm	6.4 mm	5.4 mm

Table S10. *Low- and high- frequency data. Position where the amplitude and percent signal change have dropped to 75% of maximum, expressed in millimeters (mm) away from contact with maximum activity. References that did not reach 75% of maximum is indicated with > 28 mm.*

Reference	ROI		
	OCC	PTL	ATL
SCA	35	53	139
BIP	38	52	196
LAP	36	56	190
CAR	35	56	166
REF0	37	55	152

Table S11. *Low frequency data. Number of electrodes with a significant contact as a function of reference montage (SCA, BIP, LAP, CAR, REF0) and anatomical region (OCC, PTL, ATL).*

Reference Montage	ROI		
	OCC	PTL	ATL
SCA	18	29	47
BIP	22	55	65
LAP	27	45	52
CAR	22	47	44
REF0	20	45	37

Table S12. *High frequency data. Number of electrodes with a significant contact as a function of reference montage (SCA, BIP, LAP, CAR, REF0) and anatomical region (OCC, PTL, ATL).*

TAL-Y (mm)	LAP vs. SCA	LAP vs. CAR	LAP vs. REF0	BIP vs. SCA	BIP vs. CAR	BIP vs. REF0
-75	*n.s.*	*n.s.*	*n.s.*	2.13, .036, 97	*n.s.*	*n.s.*
-65	2.11, .036, 156	*n.s.*	*n.s.*	2.44, .016, 149	*n.s.*	*n.s.*
-45	4.58, <.001, 306	3.19, .002, 342	3.93, <.001, 331	4.34, <.001, 326	2.95, .003, 362	3.69, <.001, 351
-35	4.01, <.001, 247	3.29, .001, 279	3.51, .001, 261	3.44, .001, 260	2.57, .001, 292	2.84, .005, 274
-15	2.19, .03, 160	*n.s.*	2.39, .018, 178	2.55, .012, 153	*n.s.*	2.78, .006, 171
-5	2.14, .034, 181	*n.s.*	*n.s.*	2.01, .046, 187	*n.s.*	*n.s.*

Table S13. *Low frequency data. Statistics for comparisons of references as a function of Y-Talairach coordinates (mm) for Z-scores. Coordinates with p < .05 are reported, with each cell reporting the t-value, p-value, and degrees of freedom, respectively. N.s. stands for not significant.*

TAL-Y (mm)	LAP vs. BIP	LAP vs. CAR	LAP vs. REF0	LAP vs. SCA	BIP vs. REF0	BIP vs. SCA	BIP vs. CAR	CAR vs. SCA
-85	2.22, .029, 92	*n.s.*	*n.s.*	*n.s.*	2.34, .021, 104	2.79, .006, 96	*n.s.*	*n.s.*
-75	2.18, .032, 92	*n.s.*	*n.s.*	*n.s.*	-3.4, .001, 101	-4.01, <.001, 97	-3.64, <.001, 95	*n.s.*
-55	2.01, .047, 127	*n.s.*	*n.s.*	*n.s.*	-2.4, .018, 125	-2.62, .01, 123	*n.s.*	*n.s.*
-45	3.04, .003, 334	3.53, <.001, 342	2.89, .004, 331	2.3, .022, 306	*n.s.*	*n.s.*	*n.s.*	*n.s.*
-35	3.15, .002, 267	2.43, .016, 279	*n.s.*	*n.s.*	-2.16, .01, 274	-3.4, .001, 260	*n.s.*	-2.52, .012, 272
-25	3.28, .001, 340	2.91, .004, 345	*n.s.*	*n.s.*	-2.08, .039, 303	-3.02, .003, 284	*n.s.*	-2.69, .008, 289
-15	3.52, .001, 177	4.07, <.001, 180	3.21, .002, 178	2.08, .039, 160	*n.s.*	*n.s.*	*n.s.*	-2.56, .011, 156
-5	3.73, <.001, 230	3.17, .002, 198	2.91, .004, 198	*n.s.*	*n.s.*	-2.35, .02, 187	*n.s.*	-2.11, .037, 155

Table S14. *Low frequency data. Statistics for comparisons of references as a function of Y-Talairach coordinates (mm) for signal. Coordinates with p < .05 are reported, with each cell reporting the t-value, p-value, and degrees of freedom, respectively. N.s. stands for not significant.*

TAL-Y (mm)	LAP vs. BIP	LAP vs. CAR	LAP vs. REF0	LAP vs. SCA	BIP vs. REF0	BIP vs. SCA	BIP vs. CAR	CAR vs. SCA	CAR vs. REF0	REF0 vs. SCA
-85	2.02, .046, 92	*n.s.*	*n.s.*	*n.s.*	-2.53, .013, 104	-3.55, .001, 96	-2.33, .022, 100	*n.s.*	*n.s.*	*n.s.*
-75	2.23, .028, 92	*n.s.*	*n.s.*	-2.72, .008, 99	-5.00, <.001, 101	-6.71, <.001, 97	-4.69, <.001, 95	-2.06, .042, 102	*n.s.*	-2.09, .039, 108
-65	2.68, .008, 157	*n.s.*	*n.s.*	*n.s.*	-2.33, .021, 151	-3.85, <.001, 149	*n.s.*	-3.24, .001, 149	*n.s.*	-2.17, .032, 150
-55	2.68, .008, 127	*n.s.*	*n.s.*	*n.s.*	-4.67, <.001, 125	-5.93, <.001, 123	-2.96, .004, 128	-3.62, <.001, 127	-2.1, .037, 129	*n.s.*
-45	4.14, <.001, 334	2.82, .005, 342	*n.s.*	*n.s.*	-4.59, <.001, 351	-6.04, <.001, 326	-2.21, .028, 362	-4.47, <.001, 334	-2.73, .007, 359	*n.s.*
-35	4.79, <.001, 267	*n.s.*	*n.s.*	-3.66, <.001, 247	-8.18, <.001, 274	-11.11, <.001, 260	-5.56, <.001, 292	-6.41, <.001, 272	-3.19, .002, 286	-3.04, .003, 254
-25	4.17, <.001, 340	2.37, .018, 345	*n.s.*	*n.s.*	-4.35, <.001, 303	-5.61, <.001, 284	-2.53, .012, 345	-3.83, <.001, 289	-2.21, .028, 308	*n.s.*
-15	4.05, <.001, 177	3.64, <.001, 180	*n.s.*	*n.s.*	-3.1, .002, 171	-3.97, <.001, 153	*n.s.*	-3.75, <.001, 156	-2.53, .012, 174	*n.s.*
-5	4.1, <.001, 230	2.74, .007, 198	2.06, .041, 198	*n.s.*	-2.11, .036 204	-4.03, <.001, 187	*n.s.*	-2.76, .007, 155	*n.s.*	*n.s.*

Table S15. *Low frequency data. Statistics for comparisons of references as a function of Y-Talairach coordinates (mm) for noise. Coordinates with p < .05 are reported, with each cell reporting the t-value, p-value, and degrees of freedom, respectively. N.s. stands for not significant.*

TAL-Y (mm)	BIP vs. SCA	LAP vs. SCA	REF0 vs. SCA	CAR vs. SCA	BIP vs. CAR	LAP vs. REF0	BIP vs. REF0
-75	*n.s.*	*n.s.*	*n.s.*	*n.s.*	2.20, .035, 33	*n.s.*	*n.s.*
-65	2.15, .036, 58	2.55, .014, 53	2.48, .018, 39	2.18, .034, 50	*n.s.*	*n.s.*	*n.s.*
-55	2.44, .018, 49	2.23, .03, 51	2.28, .027, 43	2.37, .022, 47	*n.s.*	*n.s.*	*n.s.*
-35	2.90, .005, 88	2.61, .011, 83	3.11, .003, 69	2.53, .013, 85	*n.s.*	*n.s.*	*n.s.*
-25	3.24, .002, 80	3.68, .001, 75	*n.s.*	2.96, .004, 66	*n.s.*	2.32, .024, 67	2.03, .046, 72
-15	3.14, .003, 55	3.55, .001, 53	*n.s.*	*n.s.*	*n.s.*	*n.s.*	*n.s.*
-5	2.81, .007, 48	3.48, .001, 46	*n.s.*	*n.s.*	*n.s.*	*n.s.*	*n.s.*

Table S16. *High frequency data. Statistics for comparisons of references as a function of Y-Talairach*

coordinates (mm) for Z-scores. Coordinates with p < .05 are reported, with each cell reporting the t-value, p-value, and degrees of freedom, respectively. N.s. stands for not significant.

TAL-Y (mm)	BIP vs. SCA	LAP vs. SCA	REF0 vs. SCA	CAR vs. SCA	BIP vs. CAR
-85	*n.s.*	*n.s.*	*n.s.*	2.13, .043, 26	*n.s.*
-75	2.42, .024, 24	*n.s.*	*n.s.*	*n.s.*	2.06, .047, 33
-65	*n.s.*	*n.s.*	2.27, .029, 39	*n.s.*	*n.s.*
-55	2.74, .008, 49	2.46, .017, 51	2.23, .031, 43	2.42, .019, 47	*n.s.*
-45	2.17, .032, 121	2.02, .045, 115	*n.s.*	*n.s.*	*n.s.*
-35	2.94, .004, 88	2.62, .01, 83	3.35, .001, 69	2.82, .006, 85	*n.s.*
-25	2.44, .017, 80	2.56, .012, 75	*n.s.*	2.67, .009, 66	*n.s.*
-15	2.11, .04, 55	2.41, .02, 53	*n.s.*	*n.s.*	*n.s.*
-5	2.35, .023, 48	*n.s.*	*n.s.*	*n.s.*	*n.s.*

Table S17. *High frequency data. Statistics for comparisons of references as a function of Y-Talairach coordinates (mm) for signal. Coordinates with p < .05 are reported, with each cell reporting the t-value, p-value, and degrees of freedom, respectively. N.s. stands for not significant.*

TAL-Y (mm)	BIP vs. SCA	LAP vs. SCA	CAR vs. SCA	LAP vs. REF0	BIP vs. REF0
-85	2.48, .02, 25	2.70, .012, 25	2.11, .045, 26	*n.s.*	*n.s.*
-55	*n.s.*	*n.s.*	*n.s.*	2.22, .03, 60	2.39, .02, 58
-45	*n.s.*	*n.s.*	*n.s.*	2.29, .024, 138	2.64, .009, 144
-25	*n.s.*	-2.24, .028, 75	*n.s.*	*n.s.*	*n.s.*

Table S18. *High frequency data. Statistics for comparisons of references as a function of Y-Talairach coordinates (mm) for noise. Coordinates with p < .05 are reported, with each cell reporting the t-value, p-value, and degrees of freedom, respectively. N.s. stands for not significant.*

Reference	Low frequencies		High frequencies	
	Onset	Peak	Onset	Peak
BIP (*N*=141, *N*=79)	142 ms	280 ms	128 ms	210 ms
LAP (*N*=134, *N*=73)	140 ms	276 ms	134 ms	216 ms
CAR (*N*=140, *N*=65)	144 ms	294 ms	128 ms	204 ms
REF0 (*N*=134, *N*=60)	142 ms	292 ms	128 ms	204 ms
SCA (*N*=121, *N*=49)	142 ms	300 ms	110 ms	186 ms

Table S19. *Timing in bilateral latFG. Onset and peak times for each reference montage (rows) for low- and high-frequency data. The onset is defined as the time point (ms) at which the activity reached 25% of the peak activity. Numbers in the brackets (column 1) show the number of significant contacts for low- and high-frequency data, respectively.*

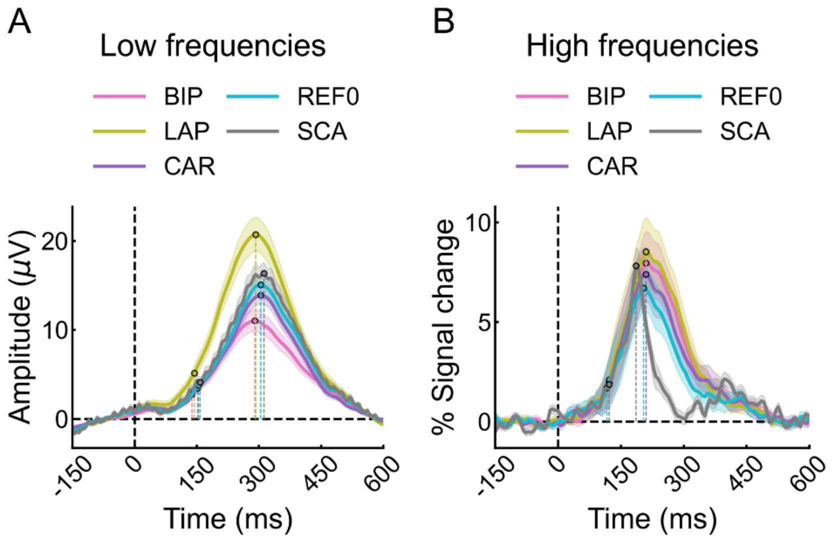

Figure S2. *Timing in bilateral ATL. Timing of face-selective activity for each reference montage for* ***(A)*** *low frequency data, and* ***(B)*** *high frequency data. Shaded area represents 95% confidence intervals (CIs). Dashed lines and dots reflect the time at which the amplitudes reached 25% of peak ("onset" phase) and peak activity.*

Reference	Low frequencies		High frequencies	
	Onset	Peak	Onset	Peak
BIP (*N*=524, *N*=113)	138 ms	290 ms	122 ms	210 ms
LAP (*N*=508, *N*=98)	144 ms	292 ms	122 ms	210 ms
CAR (*N*=504, *N*=85)	152 ms	304 ms	122 ms	210 ms
REF0 (*N*=452, *N*=70)	154 ms	304 ms	116 ms	204 ms
SCA (*N*=386, *N*=151)	158 ms	312 ms	122 ms	186 ms

Table S20. *Timing in bilateral ATL. Onset and peak times for each reference montage (rows) for low- and high-frequency data. The onset is defined as the time point (ms) at which the activity reached 25% of the peak. Numbers in the brackets (column 1) show the number of significant contacts for low- and high-frequency data, respectively.*

REF.	N	%	SCA	BIP	LAP	REF0	CAR
SCA	142 (35)	17%	*NA*	23.5, <.001	24.2, <.001	3.7, .053	17.3, < .001
BIP	206 (55)	21%	23.5, < .001	*NA*	.01, .945	8.6, .003	.5, .481
LAP	207 (56)	22%	24.2, < .001	.01, .945	*NA*	9.0, .003	.6, .438
REF0	166 (41)	18%	3.7, .053	8.6, .003	9.0, .003	*NA*	5.0, .026
CAR	196 (47)	20%	17.3, < .001	.5, .481	.6, .438	5.0, .026	*NA*

Table S21. *Low frequency data. Descriptives (columns 2 - 3) and statistics (columns 4 - 8) for white matter (WM) contact as a function of reference montage. Values in brackets of 2nd column indicate the number of subjects with at least one significant WM contact. Values in column 3 indicate the percentage of significant WM contacts relative to the number of significant grey matter contacts. Statistical comparisons report the t- and p-values, respectively, from independent t-tests.*

	N **WM reference**	***N*** **GM reference**	**% with WM reference**
BIP	110	96	53 %
LAP	74	133	36 %

Table 22. *Low frequency data. Descriptives for BIP and LAP white matter contacts as a function whether the reference contact was in the white matter or the grey matter. For LAP, both references had to be in the WM to be considered a WM reference.*

REF.	N	%	SCA	BIP	LAP	REF0	CAR
SCA	77 (16)	25%	*NA*	61.1, < .001	64.0, < .001	83.6, <. 001	70.1, < .001
BIP	22 (13)	7%	61.1, < .001	*NA*	.1, .829	2.7, .104	.4, .508
LAP	21 (15)	7%	64.0, < .001	.1, .829	*NA*	2.0, .157	.2, .655
REF0	15 (12)	6%	83.6, < .001	2.7, .104	2.0, .157	*NA*	.9, .332
CAR	19 (13)	7%	70.1, < .001	.4, .508	.2, .655	.9, .332	*NA*

Table S23. *High frequency data. Descriptives (columns 2 - 3) and statistics (columns 4 - 8) for white matter (WM) contact as a function of reference montage. Values in brackets of 2nd column indicate the number of subjects with at least one significant WM contact. Values in column 3 indicate the percentage of significant WM contacts relative to the number of significant grey matter contacts. Statistical comparisons report the t- and p-values, respectively, from independent t-tests.*

	N **WM reference**	***N*** **GM reference**	**% with WM reference**
**BIP**	6	16	27%
**LAP**	1	20	5%

Table 24. *High frequency data. Descriptives for BIP and LAP white matter contacts as a function whether the reference contact was in the white matter or the grey matter. For LAP, both references had to be in the WM to be considered a WM reference.*

References

Allison, T., Ginter, H., McCarthy, G., Nobre, A. C., Puce, A. I. N. A., Luby, M. A. R. I. E., & Spencer, D. D. (1994a). Face recognition in human extrastriate cortex. *Journal of neurophysiology*, *71*(2), 821-825.

Allison, T., McCarthy, G., Nobre, A., Puce, A., & Belger, A. (1994b). Human extrastriate visual cortex and the perception of faces, words, numbers, and colors. *Cerebral cortex*, *4*(5), 544-554.

Allison, T., Puce, A., Spencer, D. D., & McCarthy, G. (1999). Electrophysiological studies of human face perception. I: Potentials generated in occipitotemporal cortex by face and non-face stimuli. *Cerebral cortex*, *9*(5), 415-430.

Allison, T., Puce, A., & McCarthy, G. (2002). Category-sensitive excitatory and inhibitory processes in human extrastriate cortex. *Journal of neurophysiology*, *88*(5), 2864-2868.

Agam, Y., Liu, H., Papanastassiou, A., Buia, C., Golby, A. J., Madsen, J. R., & Kreiman, G. (2010). Robust selectivity to two-object images in human visual cortex. *Current Biology*, *20*(9), 872-879.

Barbeau, E. J., Taylor, M. J., Regis, J., Marquis, P., Chauvel, P., & Liégeois-Chauvel, C. (2008). Spatio temporal dynamics of face recognition. *Cerebral Cortex*, *18*(5), 997-1009.

Boring, M. J., Silson, E. H., Ward, M. J., Richardson, R. M., Fiez, J. A., Baker, C. I., & Ghuman, A. S. (2021). Multiple adjoining word-and face-selective regions in ventral temporal cortex exhibit distinct dynamics. *Journal of Neuroscience*, *41*(29), 6314-6327.

Boring, M. J., Richardson, R. M., & Ghuman, A. S. (2024). Interacting ventral temporal gradients of timescales and functional connectivity and their relationships to visual behavior. *Iscience*, *27*(6).

Chong, S. C., Jo, S., Park, K. M., Joo, E. Y., Lee, M. J., Hong, S. C., & Hong, S. B. (2013). Interaction between the electrical stimulation of a face-selective area and the perception of face stimuli. *NeuroImage*, *77*, 70-76.

Davidesco, I., Zion-Golumbic, E., Bickel, S., Harel, M., Groppe, D. M., Keller, C. J., ... & Malach, R. (2014). Exemplar selectivity reflects perceptual similarities in the human fusiform cortex. *Cerebral cortex*, *24*(7), 1879-1893.

Engell, A. D., & McCarthy, G. (2010). Selective attention modulates face-specific induced gamma oscillations recorded from ventral occipitotemporal cortex. *Journal of Neuroscience*, *30*(26), 8780-8786.

Engell, A. D., & McCarthy, G. (2011). The relationship of gamma oscillations and face-specific ERPs recorded subdurally from occipitotemporal cortex. *Cerebral cortex*, *21*(5), 1213-1221.

Engell, A. D., & McCarthy, G. (2014a). Repetition suppression of face-selective evoked and induced EEG recorded from human cortex. *Human brain mapping*, *35*(8), 4155-4162.

Engell, A. D., & McCarthy, G. (2014b). Face, eye, and body selective responses in fusiform gyrus and adjacent cortex: an intracranial EEG study. *Frontiers in human neuroscience*, *8*, 642.

Fisch, L., Privman, E., Ramot, M., Harel, M., Nir, Y., Kipervasser, S., ... & Malach, R. (2009). Neural “ignition”: enhanced activation linked to perceptual awareness in human ventral stream visual cortex. *Neuron*, *64*(4), 562-574.

Grossman, S., Gaziv, G., Yeagle, E. M., Harel, M., Mégevand, P., Groppe, D. M., ... & Malach, R. (2019). Convergent evolution of face spaces across human face-selective neuronal groups and deep convolutional networks. *Nature communications*, *10*(1), 4934.

Hagen, S., Jacques, C., Maillard, L., Colnat-Coulbois, S., Rossion, B., & Jonas, J. (2020). Spatially dissociated intracerebral maps for face-and house-selective activity in the human ventral occipito-temporal cortex. *Cerebral Cortex*, *30*(7), 4026-4043.

Hagen, S., Lochy, A., Jacques, C., Maillard, L., Colnat-Coulbois, S., Jonas, J., & Rossion, B. (2021). Dissociated face-and word-selective intracerebral responses in the human ventral occipito-temporal cortex. *Brain Structure and Function*, *226*(9), 3031-3049.

Halgren, E., Baudena, P., Heit, G., Clarke, M., & Marinkovic, K. (1994). Spatio-temporal stages in face and word processing. 1. Depth recorded potentials in the human occipital and parietal lobes. *Journal of Physiology-Paris*, *88*(1), 1-50.

Halgren, E., Baudena, P., Heit, G., Clarke, M., Marinkovic, K., & Chauvel, P. (1994). Spatio-temporal stages in face and word processing. 2. Depth-recorded potentials in the human frontal and Rolandic cortices. *Journal of Physiology-Paris*, *88*(1), 51-80.

Hamamé, C. M., Vidal, J. R., Perrone-Bertolotti, M., Ossandón, T., Jerbi, K., Kahane, P., ... & Lachaux, J. P. (2014). Functional selectivity in the human occipitotemporal cortex during natural vision: Evidence from combined intracranial EEG and eye-tracking. *Neuroimage*, *95*, 276-286.

Jacques, C., Rossion, B., Volfart, A., Brissart, H., Colnat-Coulbois, S., Maillard, L., & Jonas, J. (2020). The neural basis of rapid unfamiliar face individuation with human intracerebral recordings. *Neuroimage*, *221*, 117174.

Jacques, C., Jonas, J., Colnat-Coulbois, S., Maillard, L., & Rossion, B. (2022). Low and high frequency intracranial neural signals match in the human associative cortex. *Elife*, *11*, e76544.

Jacques, C., Witthoft, N., Weiner, K. S., Foster, B. L., Rangarajan, V., Hermes, D., ... & Grill-Spector, K. (2016). Corresponding ECoG and fMRI category-selective signals in human ventral temporal cortex. *Neuropsychologia*, *83*, 14-28.

Jonas, J., Jacques, C., Liu-Shuang, J., Brissart, H., Colnat-Coulbois, S., Maillard, L., & Rossion, B. (2016). A face-selective ventral occipito-temporal map of the human brain with intracerebral potentials. *Proceedings of the National Academy of Sciences*, *113*(28), E4088-E4097.

Kadipasaoglu, C. M., Conner, C. R., Whaley, M. L., Baboyan, V. G., & Tandon, N. (2016). Category-selectivity in human visual cortex follows cortical topology: a grouped icEEG study. *PloS one*, *11*(6), e0157109.

Kadipasaoglu, C. M., Conner, C. R., Baboyan, V. G., Rollo, M., Pieters, T. A., & Tandon, N. (2017). Network dynamics of human face perception. *PLoS One*, *12*(11), e0188834.

Keller, C. J., Davidesco, I., Megevand, P., Lado, F. A., Malach, R., & Mehta, A. D. (2017).

Tuning face perception with electrical stimulation of the fusiform gyrus. *Human brain mapping*, *38*(6), 2830-2842.

Klopp, J., Marinkovic, K., Chauvel, P., Nenov, V., & Halgren, E. (2000). Early widespread cortical distribution of coherent fusiform face selective activity. *Human brain mapping*, *11*(4), 286-293.

Kuzovkin, I., Vidal, J. R., Perrone-Bertolotti, M., Kahane, P., Rheims, S., Aru, J., ... & Vicente, R.
(2020). Identifying task-relevant spectral signatures of perceptual categorization in the human cortex. *Scientific reports*, *10*(1), 7870.

Lachaux, J. P., George, N., Tallon-Baudry, C., Martinerie, J., Hugueville, L., Minotti, L., ... & Renault, B. (2005). The many faces of the gamma band response to complex visual stimuli. *Neuroimage*, *25*(2), 491-501.

Li, Y., Richardson, R. M., & Ghuman, A. S. (2019). Posterior fusiform and midfusiform contribute to distinct stages of facial expression processing. *Cerebral Cortex*, *29*(7), 3209-3219.

Matsuo, T., Kawasaki, K., Kawai, K., Majima, K., Masuda, H., Murakami, H., ... & Hasegawa, I. (2015).
Alternating zones selective to faces and written words in the human ventral occipitotemporal cortex. *Cerebral Cortex*, *25*(5), 1265-1277.

McCarthy, G., Puce, A., Belger, A., & Allison, T. (1999). Electrophysiological studies of human face perception. II: Response properties of face-specific potentials generated in occipitotemporal cortex. *Cerebral cortex*, *9*(5), 431-444.

Miller, K. J., Hermes, D., Pestilli, F., Wig, G. S., & Ojemann, J. G. (2017). Face percept formation in human ventral temporal cortex. *Journal of neurophysiology*, *118*(5), 2614-2627.

Miller, K. J., Schalk, G., Hermes, D., Ojemann, J. G., & Rao, R. P. (2016). Spontaneous decoding of the timing and content of human object perception from cortical surface recordings reveals complementary information in the event-related potential and broadband spectral change. *PLoS computational biology*, *12*(1), e1004660.

Mundel, T., Milton, J. G., Dimitrov, A., Wilson, H. W., Pelizzari, C., Uftring, S., ... & Towle, V. L. (2003). Transient inability to distinguish between faces: electrophysiologic studies. *Journal of clinical neurophysiology*, *20*(2), 102-110.

Murphey, D. K., Maunsell, J. H., Beauchamp, M. S., & Yoshor, D. (2009). Perceiving electrical stimulation of identified human visual areas. *Proceedings of the National Academy of Sciences*, *106*(13), 5389-5393.

Nobre, A. C., Allison, T., & McCarthy, G. (1994). Word recognition in the human inferior temporal lobe. *Nature*, *372*(6503), 260-263.

Norman, Y., Yeagle, E. M., Khuvis, S., Harel, M., Mehta, A. D., & Malach, R. (2019). Hippocampal sharp-wave ripples linked to visual episodic recollection in humans. *Science*, *365*(6454), eaax1030.

Parvizi, J., Jacques, C., Foster, B. L., Withoft, N., Rangarajan, V., Weiner, K. S., & Grill-Spector, K. (2012). Electrical stimulation of human fusiform face-selective regions distorts face perception. *Journal of Neuroscience*, *32*(43), 14915-14920.

Privman, E., Fisch, L., Neufeld, M. Y., Kramer, U., Kipervasser, S., Andelman, F., ... & Malach, R. (2011). Antagonistic relationship between gamma power and visual evoked potentials revealed in human visual cortex. *Cerebral Cortex*, *21*(3), 616-624.

Privman, E., Nir, Y., Kramer, U., Kipervasser, S., Andelman, F., Neufeld, M. Y., ... & Malach, R. (2007). Enhanced category tuning revealed by intracranial electroencephalograms in high-order human visual areas. *Journal of Neuroscience*, *27*(23), 6234-6242.

Puce, A., Allison, T., & McCarthy, G. (1999). Electrophysiological studies of human face perception. III: Effects of top-down processing on face-specific potentials. *Cerebral cortex*, *9*(5), 445-458.

Puce, A., Allison, T., Spencer, S. S., Spencer, D. D., & McCarthy, G. (1997). Comparison of cortical activation evoked by faces measured by intracranial field potentials and functional MRI: two case studies. *Human brain mapping*, *5*(4), 298-305.

Rangarajan, V., Hermes, D., Foster, B. L., Weiner, K. S., Jacques, C., Grill-Spector, K., & Parvizi, J. (2014). Electrical stimulation of the left and right human fusiform gyrus causes different effects in conscious face perception. *Journal of Neuroscience*, *34*(38), 12828-12836.

Rangarajan, V., Jacques, C., Knight, R. T., Weiner, K. S., & Grill-Spector, K. (2020). Diverse temporal dynamics of repetition suppression revealed by intracranial recordings in the human ventral temporal cortex. *Cerebral Cortex*, *30*(11), 5988-6003.

Rangarajan, V., & Parvizi, J. (2016). Functional asymmetry between the left and right human fusiform gyrus explored through electrical brain stimulation. *Neuropsychologia*, *83*, 29-36.

Rosburg, T., Ludowig, E., Dümpelmann, M., Alba-Ferrara, L., Urbach, H., & Elger, C. E. (2010). The effect of face inversion on intracranial and scalp recordings of event-related potentials. *Psychophysiology*, *47*(1), 147-157.

Sanada, T., Kapeller, C., Jordan, M., Grünwald, J., Mitsuhashi, T., Ogawa, H., ... & Guger, C. (2021). Multi-modal mapping of the face selective ventral temporal cortex–a group study with clinical implications for ECS, ECoG, and fMRI. *Frontiers in Human Neuroscience*, *15*, 616591.

Sato, W., Kochiyama, T., Uono, S., Matsuda, K., Usui, K., Inoue, Y., & Toichi, M. (2014). Rapid, high-frequency, and theta-coupled gamma oscillations in the inferior occipital gyrus during face processing. *Cortex*, *60*, 52-68.

Schalk, G., Kapeller, C., Guger, C., Ogawa, H., Hiroshima, S., Lafer-Sousa, R., ... & Kanwisher, N. (2017). Facephenes and rainbows: Causal evidence for functional and anatomical specificity of face and color processing in the human brain. *Proceedings of the National Academy of Sciences*, *114*(46), 12285-12290.

Schrouff, J., Raccah, O., Baek, S., Rangarajan, V., Salehi, S., Mourão-Miranda, J., ... & Parvizi, J. (2020). Fast temporal dynamics and causal relevance of face processing in the human temporal cortex. *Nature Communications*, *11*(1), 656.

Schwartz, E., Alreja, A., Richardson, R. M., Ghuman, A., & Anzellotti, S. (2023). Intracranial electroencephalography and deep neural networks reveal shared substrates for representations of face identity and expressions. *Journal of Neuroscience*, *43*(23), 4291-4303.

Seeck, M., Michel, C. M., Mainwaring, N., Cosgrove, R., Blume, H., Ives, J., ... & Schomer, D. L. (1997). Evidence for rapid face recognition from human scalp and intracranial electrodes. *Neuroreport*, *8*(12), 2749-2754.

Tanji, K., Iwasaki, M., Nakasato, N., & Suzuki, K. (2012). Face specific broadband electrocorticographic spectral power change in the rhinal cortex. *Neuroscience letters*, *515*(1), 66-70.
